# Homo-Clavulyne: Evaluation of a One-Atom Ring Expansion Strategy to Stabilize the Nine-Membered Ring Enediyne Biosynthetic Precursor

**DOI:** 10.1055/a-2951-5568

**Published:** 2026-09-14

**Authors:** Lande Hu, Myungeun Jeong, Mohammad R. Seyedsayamdost, Erik J. Sorensen

**Affiliations:** Department of Chemistry, Princeton University, Princeton, New Jersey, United States

**Keywords:** clavulynes, homo-clavulyne enediyne, Bergman cycloaromatization, Sonogashira coupling, Kobayashi borylation, Nozaki–Hiyama–Kishi cyclization

## Abstract

*Streptomyces clavuligerus* biosynthesizes a nine-membered ring enediyne that spontaneously cycloaromatizes to a 1,4-benzenoid diradical enroute to the clavulynes. Since nature also produces several isolable and exceedingly cytotoxic metabolites featuring 10-membered ring enediynes, we reasoned that a ring-expanded “homo-clavulyne” enediyne might be accessible by the concepts and methods of organic synthesis, isolable, and useful as a new molecular warhead with therapeutic applications. The design of a 10-membered ring homo-clavulyne enediyne and our circumstantial evidence for its existence are described herein.

## GRAPHICAL ABSTRACT

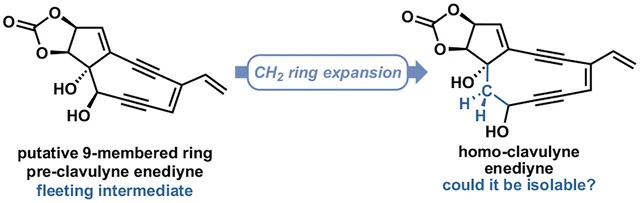

## Introduction

When the Bergman laboratory presented chemical evidence for the intermediacy of 1,4-benzenoid diradicals in thermal isomerizations of *cis*-1,5-diyn-3-enes (Fig. 1),^1^ the chemical community was unaware that nature had already evolved a family of exceedingly cytotoxic natural products that harness this chemistry to effect cleavages of double-stranded DNA and cell death. The same type of conjugated six-carbon unit at the heart of Bergman’s pioneering studies is also a prominent structural element in calicheamicin γ^I^,^2^ esperamicin,^3^ dynemicin A,^4^ maduropeptin,^5^ and other natural products (not shown)^6^ that bind to DNA and may be triggered to undergo analogous cycloaromatizations to a 1,4-benzene diradical capable of abstracting hydrogen atoms from the phosphate backbone of DNA. Neocarzinostatin is structurally distinct from these aforementioned natural products but can be induced to undergo an analogous Myers–Saito cyclization to a lethal diradical that also abstracts hydrogen atoms from DNA and triggers apoptosis.^7^ These discoveries were captivating and stimulated a great deal of research in the area of organic synthesis, with a particular focus on the context dependence of Bergman cycloaromatization chemistry, for example, correlations between molecular strain and c–d distances with proclivities toward cycloaromatization,^8^ and the therapeutic potential of the enediyne antitumor antibiotics and novel compounds derived from them (e.g., Mylotarg).^9^

Some members of this unique class of natural products, for example, sporolides A and B,^10^ the cyanonsporasides,^11^ fijiolide B,^12^ the amycolamycins,^13^ and the clavulynes,^14^ originate from enediyne precursors that are too unstable to be isolated.^15^ The clavulynes, recently described by Seyedsayamdost and coworkers,^14^ are cryptic enediyne-derived metabolites produced by *Streptomyces clavuligerus*. These metabolites arise from a fleeting nine-membered ring enediyne **1** (Scheme 1a) that cannot be isolated in laboratory settings. The assumption is that *S. clavuligerus* biosynthesizes the pre-clavulyne enediyne **1** for offensive purposes and that this reactive, unstable compound undergoes a spontaneous Bergman cyclization to a cytotoxic 1,4-benzenoid diradical (**2**), giving rise to clavulyne E (**3**) by hydrogen atom transfers to the diradical and the related monohalogenated clavulynes by the Perrin–O’Connor reaction.^16^

To date, there are at least eight structurally distinct natural products with the enediyne array confined to an isolable and relatively stable 10 membered ring. On this basis, we hypothesized that a 10-membered ring ‘homo-clavulyne’ enediyne (e.g., **4** and **5**, Scheme 1b) might be accessible by organic synthesis and possibly also isolable and therapeutically useful. A one-atom extension may seem to be a minor structural modification, yet numerous examples have demonstrated that a difference of a single atom can induce substantial changes in chemical and biological properties.^17^ The testing of this hypothesis and our initial effort to stabilize the fugitive pre-clavulyne enediyne by a one-atom ring expansion are described herein.

At the outset, our thinking was influenced by the broad applicability of the Sonogashira cross-coupling method^18^ to the problem of constructing enediyne arrays, as well as by the successful cyclizations that the groups of Kende,^19^ Danishefsky,^20^ and Nicolaou^21^ had achieved in their syntheses of the challenging molecular architecture of calicheamicin γ^I^. In principle, the 10-membered rings in homo-clavulynes **4** and **5** could arise by a strategy comprising a convergent Sonogashira coupling (e.g., between alkyne **6** and vinyl bromide **7**) and some type of intramolecular addition of an alkyne-based nucleophile to a carbonyl group. These potential bond constructions seemed reasonable and yet the highly substituted cyclopentene **6**, with its three contiguous oxygen-bearing stereocenters, would still be challenging to synthesize in a concise fashion. As matters transpired, we evaluated three distinct pathways for synthesizing compound **6** (Supporting Information) before settling on an approach featuring a 1,4-borylation/oxidation. During this undertaking, *ent*-homo-clavulyne **5** became our preferred target for synthesis only because we detected a stereochemical correspondence between key intermediate **6** and a known derivative of D-ribose. We also knew that a successful synthesis of **5** could be easily adapted to a synthesis of the enantiomeric homo-clavulyne **4** if desired.

In fact, D-ribose derivative **8** (Scheme 2a) is commercially available, relatively inexpensive, and possesses a *cis*-fused bicyclic framework that could be leveraged to control the stereochemical outcome of the eventual 1,4-borylation (vide infra). α-Iodination of cyclopentenone **8** afforded iodoenone **9** in 86% yield and was followed by standard Sonogashira coupling with trimethylsilylacetylene to give the alkynyl enone **10** in 82% yield.^18^ The two-carbon extension of the carbonyl group in **10** presented the first challenge in our synthesis. Several methods, including Horner–Wadsworth–Emmons, Peterson olefination, and aldol addition with lithio ethyl acetate followed by β-hydroxy elimination, failed to furnish the desired ester **11**. To our delight, a Wittig olefination using stabilized phosphorane **12** in refluxing toluene afforded **11** in 69% yield.

At this stage, we needed to achieve a site- and stereoselective hydration of the newly constructed electron-deficient alkene and were drawn to the effective copper-catalyzed method developed by the Kobayashi laboratory.^22^ When catalyzed by a chiral Cu^II^-bis (hydroxymethyl)bipyridyl complex, Kobayashi’s β-selective hydration of 1,6-unsaturated carbonyls is enantio-selective and performs well on a range of 1,6-unsaturated carbonyl substrates.^22^ Since the rigid, *cis*-fused bicyclic structure of **11** was expected to impart substantial facial selectivity, the achiral bis(hydroxymethyl)bipyridyl ligand (**L**) was employed for this reaction. Following reaction optimization (Supporting Information), we were pleased to find that the Cu^II^-catalyzed 1,4-borylation of compound **11** in degassed, wet tetrahydrofuran (THF) under exclusion of light, followed by in situ oxidation of the resulting organoborane, produced alcohol **13** in 65% yield on a gram scale. To set the stage for the key Sonogashira cross-coupling, compound **13** was converted to terminal alkyne **14** via compound **6** by the two-step reaction sequence shown. Notably, trimethylsilyl deprotection of the alkyne in **13** with tetra-*n*-butyl-ammonium fluoride resulted in decomposition in absence of acetic acid, possibly due to the degradation of the β-hydroxy functionality through retro-aldol or E1cB elimination under basic conditions. To suppress this degradation pathway in subsequent transformations, the alcohol was protected as the corresponding *tert*-butyldimethylsilyl ether to afford **14** as the alkyne coupling fragment. We had hoped that vinyl bromide **7**, bearing a pendant ethenyl group, could serve as a suitable coupling partner for the Sonogashira reaction (Scheme 2b). Unfortunately, however, the outcome we observed suggests that the initially formed organopalladium(II) intermediate **15** isomerizes to **17** prior to transmetalation and this isomerization is thermodynamically favored (Supporting Information). Consequently, vinyl bromide **18b** was prepared in one step from the known alcohol **18a** as an alternative coupling fragment,^23^ equipped with a protected alcohol as a useful functional group handle.

While coupling partners **14** and **18** were in hand, the pivotal Sonogashira reaction required extensive screening to find the most optimal conditions (Supporting Information). Ultimately, we found that conducting this coupling in strictly degassed, anhydrous DMF with exclusion of light and in presence of dual base additives minimizes substrate decomposition and undesired Glaser homocoupling, affording enediyne **19** in 74% yield. In the wake of this fragment coupling, the silyl-protected alkyne was converted to the corresponding iodoalkyne **20a** and also the terminal alkyne **20b** using AgNO_3_ catalysis in preparation for the efforts to construct the desired homo-clavulyne 10-membered ring.^24,25^

The two options we favored for achieving ring formation required the seemingly straightforward conversion of the ethyl esters in compounds **20a** and **20b** to aldehydes. We first attempted the complete reductions of the esters in **20a**/**20b** to primary alcohols, followed by a separate step of Dess–Martin oxidation to acquire the corresponding aldehydes. However, the resulting aldehydes underwent rapid decomposition during column chromatography on silica, deactivated silica, alumina, or Florisil, presumably via facile E1cB eliminations of the β-silyloxy substituents and secondary degradation pathways. Unfortunately, all other efforts, including Piancatelli–Margarita, Stahl, Parikh–Doering, Albright–Goldman, Ley–Griffith, Swern, and Fétizon oxidations, failed to produce the desired aldehydes (Supporting Information). Ultimately, we found that diisobutylaluminum hydride (DIBAL-H) mediated partial reductions of esters **20a** and **20b** were successful at −78 °C and afforded the sensitive aldehydes **21a** and **21b**, respectively, as stable, isolable compounds after a Fieser workup of the reactions (Scheme 3).^26^ In this fashion, the desired aldehydes were produced cleanly with no sign of unreacted starting material and only trace amounts of overreduction products.

With the desired cyclization substrates finally in hand, a one-carbon homologue of the putative pre-clavulyne enediyne **1** was nearly within reach. Could we generate a homo-clavulyne framework in the course of an intramolecular carbonyl addition and would the resulting adduct be isolable and relatively stable? We examined several methods for generating acetylide nucleophiles from **21a** or **21b** (Supporting Information). Deprotonation of the alkynyl hydrogen on **21b** with potassium bis(trimethylsilyl)amide or ^*t*^BuLi resulted in full decomposition of the aldehyde likely due to the competing deprotonation of the α-hydrogens. Carreira’s mild conditions for generating Zn^II^-acetylides using stoichiometric Zn(OTf)_2_ and NEt_3_ also led to decomposition of the aldehyde,^27^ presumably for the same reason. Eventually, we found that a Nozaki–Hiyama–Kishi reaction^28^ on substrate **21a** using catalytic NiCl_2_ and a large excess of CrCl_2_ in strictly deoxygenated and anhydrous THF generated a putative Cr^III^-acetylide that added intramolecularly to the aldehyde. We hoped and even expected to isolate 10-membered ring enediyne **23** from this reaction but could infer only that this compound formed as a transient intermediate enroute to the cycloaromatized product **25**, which we isolated after workup of the reaction. Compound **23** (or its alkoxide precursor **22**) underwent a premature Bergman cycloaromatization and the resulting benzenoid diradical **24** abstracted hydrogen atoms, presumably from THF, leading to the polycyclic benzene **25** as a 1:1 mixture of benzylic alcohol epimers. These inseparable epimers were isolated together in an overall yield of 50% from iodoalkyne ester **20a**, while the remaining of the mass balance was attributed to **21b** via inefficient acetylide addition and uncontrolled radical degradation.

The clavulynes are cryptic gene products that originate from a putative, unstable nine-membered ring enediyne. Since nature also biosynthesizes several 10-membered ring enediynes that may be isolated and even produced in the laboratory by chemical synthesis, we reasoned that a 10-membered ring homo-clavulyne might also be stable, accessible by synthesis, and available for efforts to develop its therapeutic potential. In fact, an appropriate orchestration of Wittig olefination, regio- and stereoselective 1,4-borylation/oxidation, Sonogashira cross-coupling, and ring-closing Nozaki–Hiyama–Kishi reactions are capable of generating a homo-clavulyne enediyne but only as a fugitive intermediate on a path to the product of a premature Bergman cycloaromatization. Like the pre-clavulyne enediyne **1** that inspired it, the homo-clavulyne enediyne is elusive and too close to the margins of chemical stability. Despite the unexpected result, the key strategy of Sonogashira cross-coupling, followed by intramolecular Nozaki–Hiyama–Kishi reaction leading to a fleeting benzenoid diradical intermediate analogous to **2** can be applicable to the biomimetic synthesis of clavulynes themselves. This work is part of a larger, ongoing effort to design and produce clavulyne-inspired compounds with improved stability and potential utility as starting points for the development of enediynes or latent enediynes with genuine therapeutic value.

All reactions were performed in oven-dried or flame-dried glassware under N_2_ by standard Schlenk techniques with a dry N_2_/vacuum manifold unless otherwise indicated. CuI was purified by washing with refluxing THF in Soxhlet extractor and stored under N_2_. All other reagents were purchased and used as received. Molecular sieves (4 Å, beads, 8–12 mesh) were activated in vacuum oven overnight before using. All solvents used were HPLC grade and dried by running through activated alumina columns or from Sure/Seal^™^ or AcroSeal^™^ bottles. Where indicated as strictly deoxygenated, THF was freshly distilled from sodium/benzophenone before use. Reactions were monitored via analytical thin-layer chromatography (TLC) conducted on Merck Silica gel 60 F254 plates containing a fluorescent indicator, which were visualized with iodine, ceric ammonium molybdate, KMnO_4_, *p*-anisaldehyde stain and/or UV light. Compounds were purified by flash column chromatography on Silicycle SiliaFlash P60 (230–400 mesh) silica gel or preparative TLC on SiliaPlate^™^ (20 × 20 cm, 1000 μm) containing a fluorescent indicator with ACS grade solvents. Where used, Celite filtering agent was Celite 545 purchased from Thermo Scientific. Nuclear magnetic resonance (NMR) spectra were obtained on 500 MHz Bruker spectrometers at 22 °C. ^1^H and ^13^C spectra were referenced to residual solvent (CDCl_3_: δ 7.26 ppm for ^1^H NMR and δ 77.16 ppm for ^13^C NMR). The multiplicities are abbreviated as follows: s = singlet, d = doublet, t = triplet, q = quartet, p = pentet, dd = doublet of doublets, ddd = doublet of doublet of doublets, dddd = doublet of doublet of doublet of doublets, dtt = doublet of triplet of triplets, td = triplet of doublets, tdd = triplet of doublet of doublets, qd = quartet of doublets, m = multiplet, and br = broad signal. Coupling constants were measured in Hz and abbreviated as J. Infrared (IR) spectra were recorded on a Nicolet 6700 FT-IR equipped with a 30,000–200 cm^−1^ diamond. High-resolution mass spectrometry (HRMS) data were obtained on an Agilent 6230 LC/TOF with liquid chromatography electrospray ionization (ESI).

### Procedures

#### Iodide 9

To a 500-mL round-bottom flask charged with a magnetic stir bar was added **8** (5 g, 32.43 mmol, 1 equiv), iodine (16.46 g, 64.87 mmol, 2 equiv), and CH_2_Cl_2_ (162 mL, 0.2 M). The solution was cooled to 0 °C and added pyridine (3.92 mL, 48.65 mmol, 1.5 equiv) slowly, then stirred for 16 h at room temperature. The reaction was quenched with 10% aq. ascorbic acid solution (150 mL). The organic layer was separated, and the aqueous layer was extracted with CH_2_Cl_2_ (80 mL × 3). The combined organic layers were dried over anhydrous MgSO_4_ and filtered, then concentrated in vacuo. The crude residue was purified via flash chromatography (0–15 % EtOAc/hexanes) to afford iodide **9** (7.80 g, 86%) as a white solid.

**TLC** *R*_f_ = 0.36 (20% EtOAc/hexanes); all characterizations matched with previously reported literature.^29^

#### Alkynyl enone 10

To a 1000-mL round-bottom flask charged with a magnetic stir bar was added iodide **9** (7.80 g, 27.85 mmol, 1 equiv), trimethylsilylacetylene (11.6 mL, 83.55 mmol, 3 equiv), ^*i*^Pr_2_NEt (9.7 mL, 55.70 mmol, 2 equiv), and anhydrous THF (278 mL, 0.1 M). The solution was degassed by bubbling a steady stream of N_2_ for 40 min, then swiftly added Pd(PPh_3_)_4_ (321.8 mg, 0.2785 mmol, 1 mol%) and CuI (318.3 mg, 1.671 mmol, 6 mol%). The reaction was stirred for 16 h at room temperature before quenching with sat. aq. NH_4_Cl (200 mL). The organic layer was separated, and the aqueous layer was extracted with EtOAc (150 mL × 3). The combined organic layers were dried over anhydrous MgSO_4_ and filtered, then concentrated in vacuo. The crude residue was purified via flash chromatography (0–12 % EtOAc/hexanes) to afford alkyne **10** (5.72 g, 82%) as a white solid.

**TLC** *R*_f_ = 0.39 (20% EtOAc/hexanes).

^1^**H NMR** (CDCl_3_, 500 MHz) δ 7.59 (d, *J* = 2.5 Hz, 1H), 5.24 (dd, *J* = 5.6, 2.7 Hz, 1H), 4.52 (d, *J* = 5.4 Hz, 1H), 1.41 (s, 6H), 0.23 (s, 9H).

^13^**C NMR** (CDCl_3_, 126 MHz) δ 198.6, 159.3, 130.4, 115.7, 105.4, 93.9, 77.4, 76.7, 27.6, 26.3, −0.2.

**HRMS** (+ESI-TOF) *m*/*z* calcd for C_13_H_18_NaO_3_Si^+^ [M+Na]^+^: 273.0917; found 273.0913.

**FT-IR** (neat) v 2960, 2095, 1740, 1597, 1375, 1251, 1207, 1153, 1098, 1047, 999, 916, 846, 762 cm^−1^.

#### Ester 11

To a 25-ml round-bottom flask charged with a magnetic stir bar was added alkynyl enone **10** (100.0 mg, 0.400 mmol, 1 equiv), ethyl 2-(triphenylphosphoranylidene) acetate **12** (250.5 mg, 0.719 mmol, 1.8 equiv), and toluene (7 mL, 0.057 M). The solution was stirred for 18 h at 110 °C before cooling down to room temperature and concentrating in vacuo. The crude residue was purified via flash chromatography (0–5 % EtOAc/hexanes) to afford ester **11** (87.7 mg, 69%) as a white solid.

**TLC** *R*_f_ = 0.10 (10% EtOAc/hexanes).

^1^**H NMR** (CDCl_3_, 500 MHz) δ 6.60 (d, *J* = 2.3 Hz, 1H), 6.16 (s, 1H), 5.71 (dt, *J* = 5.5, 1.0 Hz, 1H), 5.17 (dd, *J* = 5.6, 2.4 Hz, 1H), 4.26 (qd, *J* = 7.1, 3.8 Hz, 2H), 1.44 (s, 3H), 1.39 (s, 3H), 1.33 (t, *J* = 7.1 Hz, 3H), 0.24 (s, 9H).

^13^**C NMR** (CDCl_3_, 126 MHz) δ 165.9, 156.6, 145.7, 128.9, 116.4, 112.7, 102.4, 96.2, 82.1, 76.8, 60.6, 27.7, 26.3, 14.4, −0.1.

**HRMS** (+ESI-TOF) *m*/*z* calcd for C_17_H_24_NaO_4_Si^+^ [M+Na]^+^: 343.1336; found 343.1344.

**FT-IR** (neat) v 2960, 2043, 1721, 1652, 1581, 1457, 1371, 1331, 1302, 1251, 1209, 1176, 1155, 1081, 1145, 1004, 846, 761, 702 cm^−1^.

#### Alcohol 13

To a 100-mL round-bottom flask charged with a magnetic stir bar was added ester **11** (1.13 g, 3.535 mmol, 1 equiv) and wet THF (35 mL, 0.1 M). The solution was degassed by bubbling a steady stream of N_2_ for 20 min, then swiftly added Cu(OAc)_2_ (128.4 mg, 0.7070 mmol, 20 mol%), Ligand **L** (183.5 mg, 0.8484 mmol, 24 mol%), and B_2_pin_2_ (1.44 g, 5.656 mmol, 1.6 equiv). The reaction was covered with aluminum foil and stirred for 6 h at room temperature. After complete consumption of the starting material, the reaction was cooled to 0 °C and slowly added NaBO_3_·4H_2_O(1.63 g, 10.60 mmol, 3 equiv) and H_2_O (24 mL, 0.15 M). The reaction was stirred for another hour at room temperature before quenching with sat. aq. NH_4_Cl (20 mL). The organic layer was separated, and the aqueous layer was extracted with EtOAc (30 mL × 3). The combined organic layers were dried over anhydrous MgSO_4_ and filtered, then concentrated in vacuo. The crude residue was purified via flash chromatography (0–15 % EtOAc/hexanes) to afford alcohol **13** (778.7 mg, 65%) as a pale yellow oil.

**TLC** *R*_f_ = 0.45 (40% EtOAc/hexanes).

^1^**H NMR** (CDCl_3_, 500 MHz) δ 5.36 (dd, *J* = 5.6, 1.3 Hz, 1H), 4.66 (d, *J* = 4.6 Hz, 1H), 4.53 (d, *J* = 5.6 Hz, 1H), 4.17 (qd, *J* = 7.1, 4.0 Hz, 2H), 3.48 (d, *J* = 16.4 Hz, 1H), 3.27 (d, *J* = 16.4 Hz, 1H), 2.10 (d, *J* = 5.4 Hz, 1H), 1.34 (d, *J* = 6.4 Hz, 6H), 1.28 (t, *J* = 7.1 Hz, 4H), 0.21 (s, 9H).

^13^**C NMR** (CDCl_3_, 126 MHz) δ 169.7, 146.5, 126.0, 112.1, 103.2, 98.2, 84.6, 84.2, 81.9, 61.2, 33.7, 27.5, 26.1, 14.3, 0.0.

**HRMS** (+ESI-TOF) *m*/*z* calcd for C_17_H_27_O_5_Si [M+H]^+^: 339.1622; found 339.1626.

**FT-IR** (neat) v 3436, 2985, 2959, 2934, 2147, 1738, 1371, 1328, 1251, 1209, 1182, 1158, 1069, 1003, 950, 844, 761, 710 cm^−1^.

#### Enyne 6

To a 100-mL round-bottom flask charged with a magnetic stir bar was added alcohol **13** (778.7 mg, 2.30 mmol, 1 equiv) and THF (23 mL, 0.1 M). The reaction was cooled to 0°C and slowly added AcOH (0.66 mL, 11.50 mmol, 5 equiv) and ^*n*^Bu_4_NF (3.45 mL, 1 M in THF, 3.45 mmol, 1.5 equiv), then stirred for 1 h at room temperature. After complete consumption of the starting material, the reaction was quenched with sat. aq. NH_4_Cl (20 mL). The organic layer was separated, and the aqueous layer was extracted with EtOAc (20 mL × 3). The combined organic layers were dried over anhydrous MgSO_4_ and filtered, then concentrated in vacuo. The crude residue was purified via flash chromatography (0–15 % EtOAc/hexanes) to afford enyne **6** (536.4 mg, 88%) as a clear oil.

**TLC** *R*_f_ = 0.26 (40% EtOAc/hexanes).

^1^**H NMR** (CDCl_3_, 500 MHz) δ 5.36 (d, *J* = 5.6 Hz, 1H), 4.69 (dt, *J* = 5.7, 1.6 Hz, 1H), 4.55 (d, *J* = 5.6 Hz, 1H), 4.17 (qd, *J* = 7.2, 3.1 Hz, 2H), 3.50 (d, *J* = 16.3 Hz, 1H), 3.30 (d, *J* = 16.3 Hz, 1H), 3.26 (s, 1H), 2.11 (d, *J* = 5.7 Hz, 1H), 1.35 (d, *J* = 4.5 Hz, 6H), 1.28 (t, *J* = 7.2 Hz, 3H).

^13^**C NMR** (CDCl_3_, 126 MHz) δ 169.5, 147.5, 125.0, 112.2, 84.9, 84.6, 84.2, 82.0, 61.3, 33.6, 27.4, 26.1, 14.3.

**HRMS** (+ESI-TOF) *m*/*z* calcd for C_14_H_18_NaO_5_^+^ [M+Na]^+^: 289.1046; found 289.1050.

**FT-IR** (neat) v 3435, 3284, 2986, 2935, 1735, 1457, 1372, 1328, 1301, 1254, 1209, 1159, 1069, 1032, 1001, 949, 872, 805 cm^−1^.

#### Enyne 14

To a 50-mL round-bottom flask charged with a magnetic stir bar was added enyne **6** (257.0 mg, 0.9651 mmol, 1 equiv), 2,6-lutidine (0.335 mL, 2.895 mmol, 3 equiv) and anhydrous CH_2_Cl_2_ (9.7 mL, 0.1 M). The reaction was cooled to 0 °C and added ^*t*^BuMe_2_SiOTf (0.443 mL, 1.930 mmol, 2 equiv) dropwise, then stirred for 30 min at 0 °C. After complete consumption of the starting material, the reaction was quenched with H_2_O (10 mL). The organic layer was separated, and the aqueous layer was extracted with CH_2_Cl_2_ (10 mL × 3). The combined organic layers were dried over anhydrous MgSO_4_ and filtered, then concentrated in vacuo. The crude residue was purified via flash chromatography (0–5 % EtOAc/hexanes) to afford enyne **14** (360.0 mg, 98%) as a clear oil.

**TLC** *R*_f_ = 0.23 (10% EtOAc/hexanes).

^1^**H NMR** (CDCl_3_, 500 MHz) δ 5.36 (d, *J* = 5.6 Hz, 1H), 4.67 (t, *J* = 1.6 Hz, 1H), 4.41 (d, *J* = 5.6 Hz, 1H), 4.16 (qd, *J* = 7.2, 2.6 Hz, 2H), 3.46 (d, *J* = 16.2 Hz, 1H), 3.30 (d, *J* = 16.1 Hz, 1H), 3.20 (s, 1H), 1.34 (d, *J* = 2.7 Hz, 6H), 1.27 (t, *J* = 7.1 Hz, 3H), 0.92 (s, 9H), 0.16 (d, *J* = 6.6 Hz, 6H).

^13^**C NMR** (CDCl_3_, 126 MHz) δ 169.6, 146.2, 125.6, 112.1, 85.6, 84.4, 84.4, 82.8, 78.3, 61.1, 33.8, 27.5, 26.3, 26.0, 18.4, 14.3, −4.4, −4.6.

**HRMS** (+ESI-TOF) *m*/*z* calcd for C_20_H_33_O_5_Si^+^ [M+H]^+^: 381.2092; found 381.2105.

**FT-IR** (neat) v 3253, 2984, 2955, 2930, 2858, 2032, 1740, 1472, 1371, 1255, 1210, 1179, 1157, 1091, 1029, 1005, 867, 837, 805, 778 cm^−1^.

#### Vinyl Bromide 18b

To a 250-mL round-bottom flask charged with a magnetic stir bar was added alcohol **18a** (1.773 g, 7.604 mmol, 1 equiv),^23 *i*^Pr_2_NEt (5.30 mL, 30.42 mmol, 4 equiv) and anhydrous CH_2_Cl_2_ (76 mL, 0.1 M). The reaction was cooled to −78 °C and added methoxymethyl chloride (1.73 mL, 22.81 mmol, 3 equiv) dropwise, then stirred for 12 h at room temperature. After complete consumption of the starting material, the reaction was cooled to 0 °C and quenched with sat. aq. NaHCO_3_ (50 mL). The organic layer was separated, and the aqueous layer was extracted with CH_2_Cl_2_ (30 mL × 3). The combined organic layers were dried over anhydrous MgSO_4_ and filtered, then concentrated in vacuo. The crude residue was purified via flash chromatography (0–2 % EtOAc/hexanes) to afford vinyl bromide **18b** (1.594 g, 76%) as a clear oil.

**TLC** *R*_f_ = 0.39 (10% EtOAc/hexanes).

^1^**H NMR** (CDCl_3_, 500 MHz) δ 6.30 (s, 1H), 4.65 (s, 2H), 4.26 (s, 2H), 3.38 (s, 3H), 0.22 (s, 9H).

^13^**C NMR** (CDCl_3_, 126 MHz) δ 134.3, 112.0, 102.3, 100.9, 95.6, 71.1, 55.8, −0.1.

**HRMS** (+ESI-TOF) *m*/*z* calcd for C_10_H_18_BrO_2_Si^+^ [M+H]^+^: 277.0254; found 277.0530.

**FT-IR** (neat) v 2957, 2897, 2142, 1250, 1152, 1104, 1063, 1035, 924, 842, 760 cm^−1^.

#### Enediyne 19

To a 50-mL Schlenk flask charged with a magnetic stir bar was added enyne **14** (288.2 mg, 0.7573 mmol, 1 equiv), vinyl bromide **18b** (209.9 mg, 0.7573 mmol, 1 equiv), ^*i*^Pr_2_NEt (0.528 mL, 3.029 mmol, 4 equiv), 2,6-lutidine (0.175 mL, 1.515 mmol, 2 equiv), activated 4 Å molecular sieves (379 mg, 50 mg/mL), and anhydrous DMF (7.6 mL, 0.1 M). The system underwent freeze–pump–thaw protocol for three cycles then backfilled with N_2_ atmosphere. Pd(PPh_3_)_4_ (43.8 mg, 37.87 μmol, 5 mol%) and CuI (43.3 mg, 0.2272 mmol, 30 mol%) in a degassed vial was added swiftly under high N_2_ outflow. The reaction was covered with aluminum foil and stirred for 16 h at room temperature, then filtered to a separatory funnel and diluted with Et_2_O (20 mL) and sat. aq. NH_4_Cl (10 mL). The organic layer was separated, and the aqueous layer was extracted with Et_2_O (20 mL × 3). The combined organic layers were washed with 5% aq. LiCl (40 mL) and brine (30 mL), then dried over anhydrous MgSO_4_, filtered, and concentrated in vacuo. The crude residue was purified via flash chromatography (0–10 % EtOAc/hexanes) to afford enediyne **19** (324.9 mg, 74%) as a pale yellow oil.

**TLC** *R*_f_ = 0.44 (20% EtOAc/hexanes).

^1^**H NMR** (CDCl_3_, 500 MHz) δ 6.05 (t, *J* = 1.7 Hz, 1H), 5.42 (d, *J* = 5.7 Hz, 1H), 4.69 (t, *J* = 1.5 Hz, 1H), 4.63 (s, 2H), 4.43 (d, *J* = 5.6 Hz, 1H), 4.15 (q, *J* = 7.1 Hz, 2H), 4.12 (d, *J* = 1.8 Hz, 2H), 3.60 (d, *J* = 16.4 Hz, 1H), 3.36 (s, 3H), 3.33 (d, *J* = 16.3 Hz, 1H), 1.34 (s, 6H), 1.26 (t, *J* = 7.1 Hz, 3H), 0.91 (s, 9H), 0.20 (s, 9H), 0.16 (d, *J* = 9.3 Hz, 6H).

^13^**C NMR** (CDCl_3_, 126 MHz) δ 169.6, 146.0, 131.4, 126.1, 115.7, 112.0, 103.1, 102.3, 95.8, 92.7, 91.9, 85.7, 84.3, 82.8, 68.3, 61.1, 55.7, 33.9, 27.6, 26.4, 26.0, 18.4, 14.3, 0.1, −4.3, −4.5.

**HRMS** (+ESI-TOF) *m*/*z* calcd for C_30_H_49_O_7_Si_2_^+^ [M+H]^+^: 577.3011; found 577.3023.

**FT-IR** (neat) v 2955, 2930, 2857, 2035, 1740, 1472, 1371, 1251, 1211, 1152, 1093, 1044, 1007, 921, 842, 778, 761 cm^−1^.

#### Iodide 20a

To an 8-mL vial charged with a magnetic stir bar was added enediyne **19** (50.0 mg, 86.67 μmol, 1 equiv), *N*-iodosuccinimide (39.0 mg, 0.1733 mmol, 2 equiv), AgNO_3_ (5.9 mg, 34.67 μmol, 40 mol%), and anhydrous DMF (1.7 mL, 0.05 M). The reaction was covered with aluminum foil and stirred for 3 h at room temperature before quenching with sat. aq. Na_2_S_2_O_3_ (2 mL). The aqueous layer was extracted with Et_2_O (5 mL × 3). The combined organic layers were washed with 5% aq. LiCl (10 mL) and brine (10 mL), then dried over anhydrous MgSO_4_, filtered, and concentrated in vacuo. The crude residue was purified via flash chromatography (0–10 % EtOAc/hexanes) to afford iodide **20a** (36.5 mg, 67%) as a pale yellow oil.

**TLC** *R*_f_ = 0.33 (20% EtOAc/hexanes).

^1^**H NMR** (CDCl_3_, 500 MHz) δ 6.15 (t, *J* = 1.8 Hz, 1H), 5.41 (dd, *J* = 5.6, 1.2 Hz, 1H), 4.70 (d, *J* = 1.6 Hz, 1H), 4.64 (s, 2H), 4.43 (d, *J* = 5.6 Hz, 1H), 4.17 (qd, *J* = 7.2, 5.7 Hz, 2H), 4.13 (d, *J* = 1.8 Hz, 2H), 3.58 (d, *J* = 16.3 Hz, 1H), 3.40 (d, 1H), 3.37 (s, 3H), 1.35 (d, *J* = 4.5 Hz, 6H), 1.28 (t, *J* = 7.2 Hz, 3H), 0.92 (s, 9H), 0.17 (d, *J* = 8.7 Hz, 6H).

^13^**C NMR** (CDCl_3_, 126 MHz) δ 169.7, 146.8, 133.3, 126.0, 115.8, 112.1, 95.9, 92.6, 92.2, 92.0, 85.7, 84.4, 82.6, 68.1, 61.1, 55.7, 34.1, 29.9, 27.6, 26.4, 26.0, 18.4, 14.4, −4.3, −4.5.

**HRMS** (+ESI-TOF) *m*/*z* calcd for C_27_H_40_IO_7_Si^+^ [M+H]^+^: 631.1583; found 631.1589.

**FT-IR** (neat) v 2930, 2857, 2007, 1738, 1472, 1372, 1256, 1213, 1154, 1080, 862, 839, 781 cm^−1^.

#### Enediyne 20b

To an 8-mL vial charged with a magnetic stir bar was added enediyne **19** (100.0 mg, 0.1733 mmol, 1 equiv), AgNO_3_ (5.9 mg, 34.67 μmol, 40 mol%), H_2_O (0.31 mL, 17.33 mmol, 100 equiv), and anhydrous acetone (1.7 mL, 0.1 M). The reaction was covered with aluminum foil and stirred for 2 h at room temperature. After complete consumption of the starting material, the reaction was purified directly via preparative TLC (20 % EtOAc/hexanes) to afford Enediyne **20b** (71.4 mg, 82%) as a pale yellow oil.

**TLC** *R*_f_ = 0.34 (20% EtOAc/hexanes).

^1^**H NMR** (CDCl_3_, 500 MHz) δ 6.01 (q, *J* = 1.8 Hz, 1H), 5.43 (d, *J* = 5.6 Hz, 1H), 4.70 (s, 1H), 4.65 (s, 2H), 4.43 (d, *J* = 5.6 Hz, 1H), 4.21–4.09 (m, 4H), 3.63 (d, *J* = 16.4 Hz, 1H), 3.38 (s, 3H), 3.36–3.31 (m, 2H), 1.34 (s, 6H), 1.26 (t, *J* = 7.1 Hz, 3H), 0.92 (s, 9H), 0.16 (d, *J* = 8.3 Hz, 6H).

^13^**C NMR** (CDCl_3_, 126 MHz) δ 169.6, 146.8, 133.1, 126.1, 114.8, 112.1, 96.0, 92.6, 92.1, 85.7, 85.0, 84.4, 82.5, 81.3, 77.4, 68.2, 61.0, 55.7, 33.8, 27.5, 26.3, 26.0, 18.4, 14.3, −4.3, −4.6.

**HRMS** (+ESI-TOF) *m*/*z* calcd for C_27_H_40_NaO_7_Si^+^ [M+Na]^+^: 527.2436; found 527.2478.

**FT-IR** (neat) v 3288, 2930, 2857, 1975, 1738, 1472, 1371, 1331, 1254, 1211, 1183, 1152, 1091, 1060, 1036, 1007, 922, 866, 838, 778 cm^−1^.

#### Polycyclic Benzene 25

To a 5-mL round-bottom flask charged with a magnetic stir bar was added iodide **20a** (11.7 mg, 18.55 μmol, 1 equiv) and anhydrous CH_2_Cl_2_ (0.37 mL, 0.05 M). The reaction was cooled to −78 °C and added DIBAL-H (0.028 mL, 1 M in PhMe, 27.83 μmol, 1.5 equiv) dropwise. (Note: dropwise addition along the side of the glass was required to prevent over reduction.) The reaction was stirred at −78 °C for 1 h. After complete consumption of the starting material, the reaction was diluted with Et_2_O (5 mL), quenched with one drop of H_2_O, one drop of 15% aq. NaOH, and two drops of H_2_O, then stirred at room temperature for 15 min. The mixture was dried over anhydrous MgSO_4_, filtered, and concentrated in vacuo as aldehyde **21a**. The crude residue was used in the next step without purification. (Note: aldehyde **21a** decomposes upon chromatographic purification.)

To an 8-mL vial charged with a magnetic stir bar was added NiCl_2_ (0.5 mg, 3.71 μmol, 20 mol%), CrCl_2_ (22.8 mg, 0.1855 mmol, 10 equiv), activated 4 Å molecular sieves (185 mg, 50 mg/mL), and strictly deoxygenated THF (2.7 mL, freshly distilled from sodium/benzophenone) under N_2_ atmosphere. A solution of crude aldehyde **21a** (10.9 mg, 18.55 μmol, 1 equiv) in strictly deoxygenated THF (1 mL) was added dropwise over 30 min. The reaction was further stirred at room temperature for 3 h, then filtered to a separatory funnel and diluted with Et_2_O (5mL) and sat. aq. NH_4_Cl (5 mL). The organic layer was separated, and the aqueous layer was extracted with Et_2_O (5 mL × 3). The combined organic layers were washed with brine (15 mL), then dried over anhydrous MgSO_4_, filtered, and concentrated in vacuo. The crude residue was purified via preparative TLC (40% EtOAc/hexanes) to afford polycyclic benzene **25** (4.3 mg, 50% over two steps, mixture of inseparable diastereomers, 1:1 d.r.) as a clear oil.

**TLC** *R*_f_ = 0.35 (40% EtOAc/hexanes).

^1^**H NMR** (CDCl_3_, 500 MHz) δ 7.45–7.36 (m, 4H), 7.29–7.26 (m, 2H), 5.28 (t, *J* = 5.0 Hz, 2H), 5.14 (d, *J* = 11.9 Hz, 2H), 4.94 (q, *J* = 5.8 Hz, 1H), 4.86 (q, *J* = 3.9 Hz, 1H), 4.71 (d, *J* = 3.4 Hz, 4H), 4.60–4.56 (m, 6H), 3.41 (d, *J* = 3.1 Hz, 6H), 2.80–2.63 (m, 4H), 1.38 (s, 6H), 1.33 (d, *J* = 11.0 Hz, 6H), 0.90 (d, *J* = 13.7 Hz, 18H), 0.29–0.22 (m, 12H).

^13^**C NMR** (CDCl_3_, 126 MHz) δ 139.3, 138.9, 138.3, 138.2, 137.0, 136.8, 136.3, 135.7, 130.9, 130.6, 127.7, 127.5, 127.5, 127.4, 124.2, 124.1, 111.9, 111.8, 96.0, 95.9, 87.2, 87.0, 85.4, 84.8, 80.3, 80.2, 77.4, 69.0, 69.0, 68.5, 68.4, 55.5, 55.5, 31.7, 31.5, 29.9, 27.9, 27.7, 26.6, 26.4, 26.0, 26.0, −3.9, −3.9, −4.6, −4.6.

**HRMS** (+ESI-TOF) *m*/*z* calcd for C_25_H_38_NaO_6_Si^+^ [M+Na]^+^: 485.2330; found 485.2335.

**FT-IR** (neat) v 3446, 2927, 2856, 1463, 1379, 1254, 1208, 1150, 1075, 1051, 920, 865, 838, 776 cm^−1^.

## Supplementary Material

1

**Supplementary Material** is available online at https://doi.org/10.1055/a-2951-5568

## Figures and Tables

**Fig. 1:**
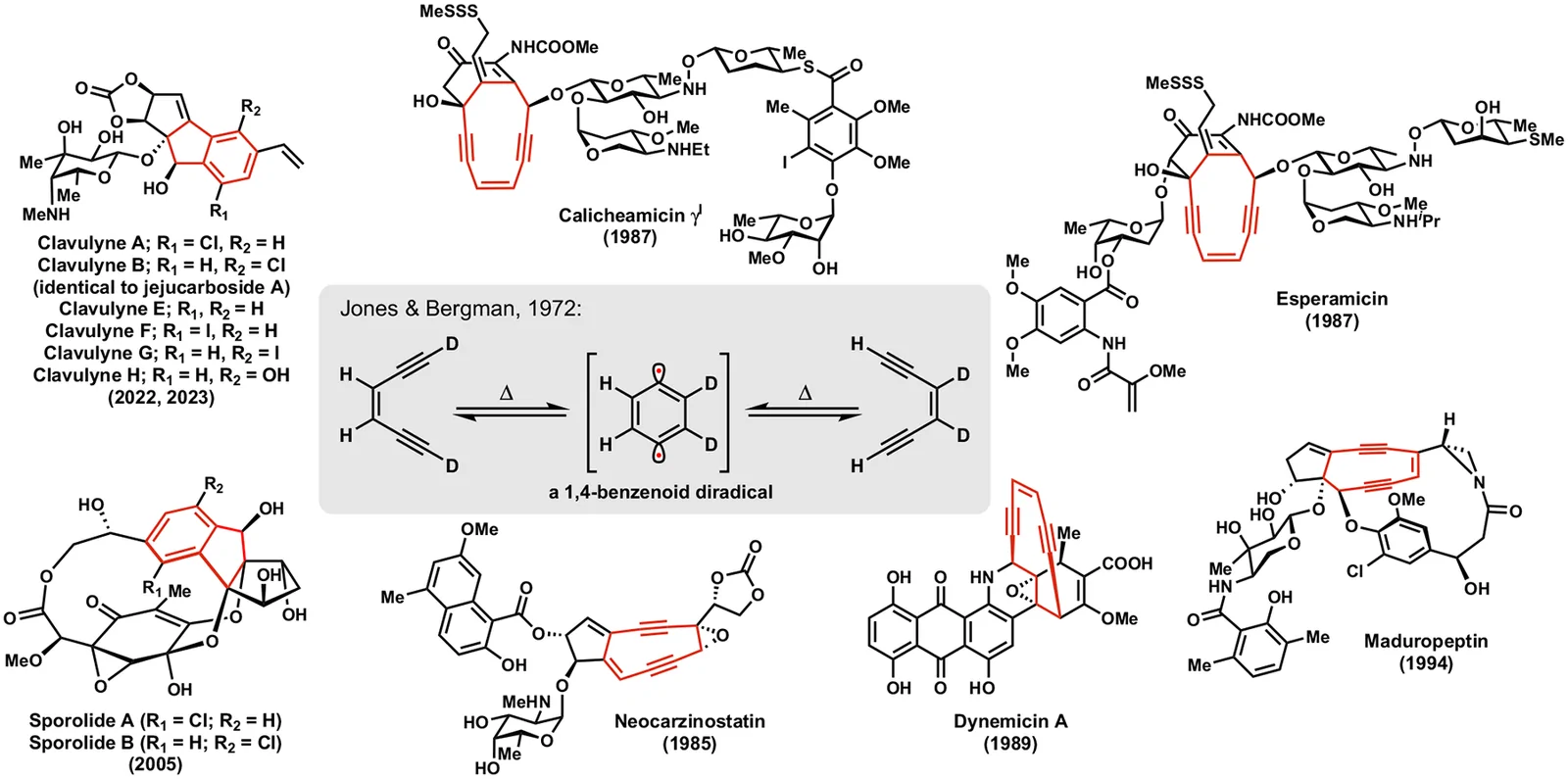
The Bergman cycloaromatization and the structures of selected members of the enediyne class of antitumor natural products.

**Scheme 1:**
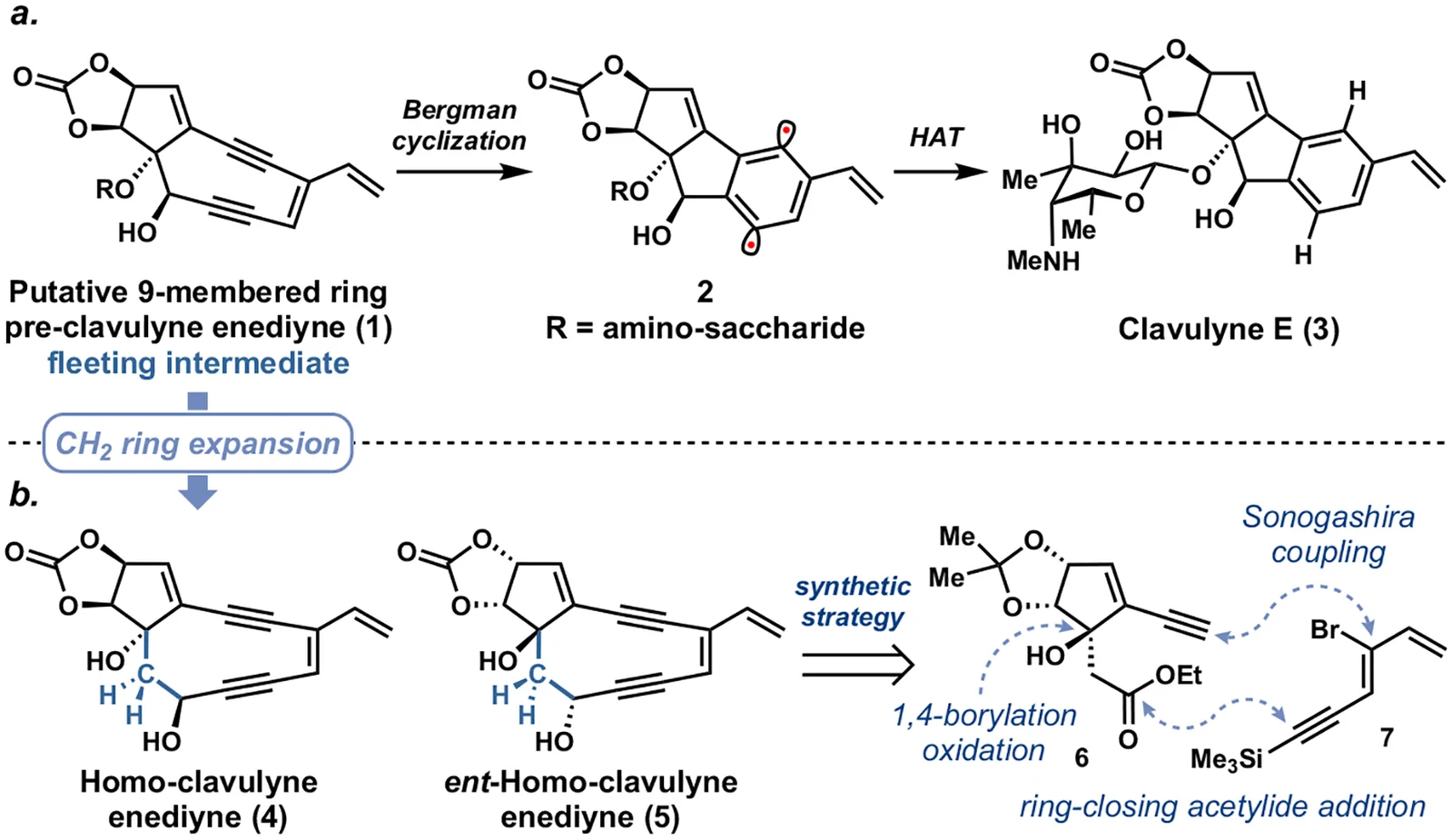
(**a**) The putative nine-membered ring pre-clavulyne enediyne (**1**), cytotoxic 1,4-benzenoid diradical (**2**) and clavulyne E (**3**). (**b**) The structures of enantiomeric 10-membered ring homo-clavulyne enediynes **4** and **5**, and our synthetic strategy.

**Scheme 2:**
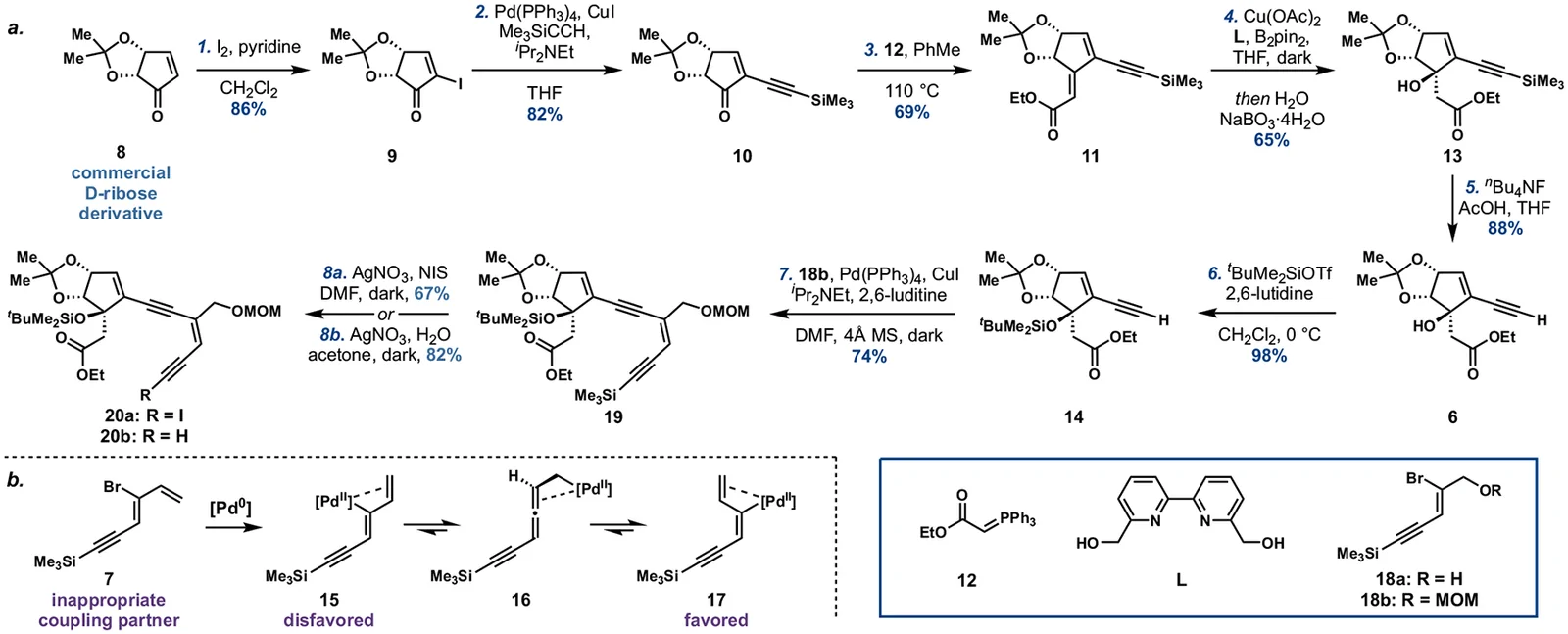
(**a**) Synthesis of potential cyclization substrates **20a** and **20b** featuring a 1,4-borylation/oxidation and a Sonogashira fragment coupling. Reagents and conditions: 1. I_2_ (2 equiv), pyridine (1.5 equiv), CH_2_Cl_2_ (0.2 M), 0 to 23°C, 16 h, 86%; 2. Pd(PPh_3_)_4_ (1 mol%), CuI (6 mol%), trimethylsilylacetylene (3 equiv), ^*i*^Pr_2_NEt (2 equiv), THF (0.1 M), 23°C, 16 h, 82%; 3. 12 (1.8 equiv), PhMe (0.057 M), 110°C, 18 h, 69%; 4. Cu(OAc)_2_ (20 mol%), L (24 mol%), B_2_pin_2_ (1.6 equiv), wet THF (0.1 M), dark, 23°C, 6 h, *then* NaBO_3_·4H_2_O (3 equiv), H_2_O (0.15 M), 0–23°C, 1 h, 65%; 5. ^*n*^Bu_4_NF (1.5 equiv), AcOH (5 equiv), THF (0.1 M), 0–23°C, 1 h, 88%; 6. ^*t*^BuMe_2_SiOTf (2 equiv), 2,6-lutidine (3 equiv), CH_2_Cl_2_ (0.1 M), 0°C, 30 min, 98%; 7. 18 (1 equiv), Pd(PPh_3_)_4_ (5 mol%), CuI (30 mol%), ^*i*^Pr_2_NEt (4 equiv), 2,6-lutidine (2 equiv), DMF (0.1 M), 4Å MS, dark, 23°C, 16 h, 74%; 8a. AgNO_3_ (40 mol%), NIS (2 equiv), DMF (0.05 M), dark, 23°C, 3 h, 67%; 8b. AgNO_3_ (40 mol%), H_2_O (100 equiv), acetone (0.1 M), dark, 23°C, 2 h, 82%. (**b**) Inappropriate coupling partner 7. B_2_pin_2_ = Bis(pinacolato)diboron; NIS = *N*-iodosuccinimide; MS = molecular sieves.

**Scheme 3:**
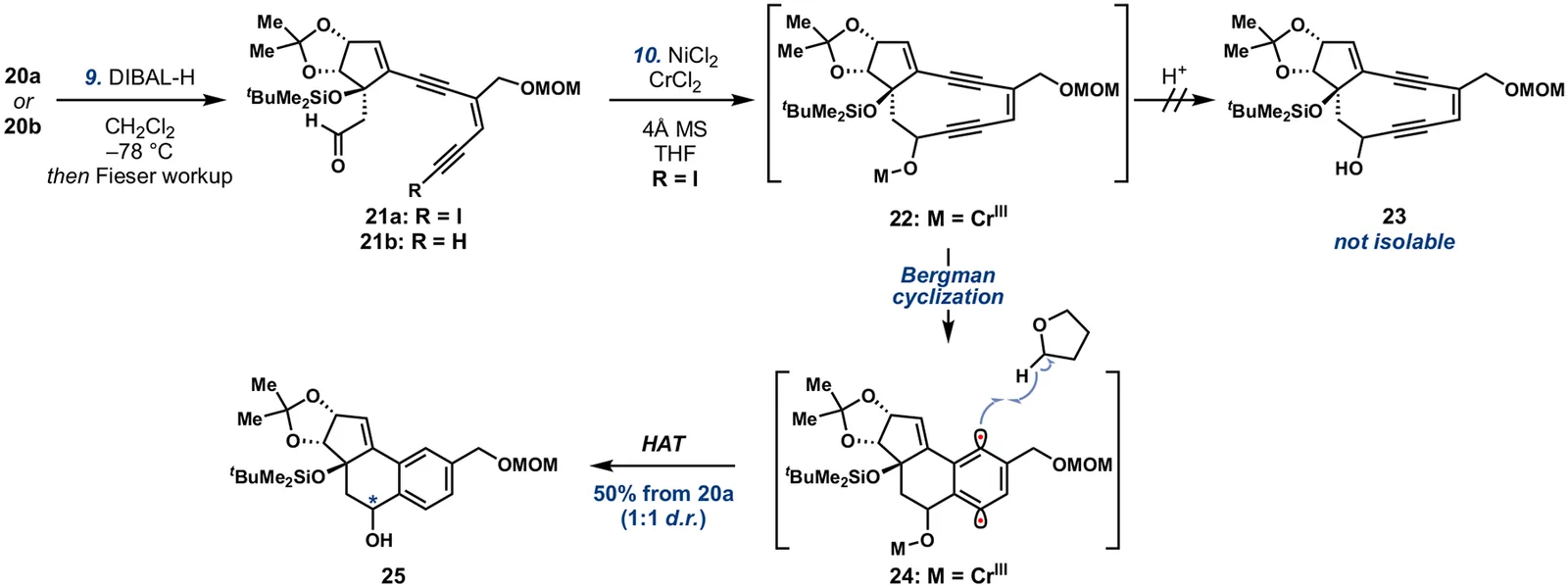
Ring-closing Nozaki–Hiyama–Kishi coupling to forge the 10-membered enediyne framework and the subsequent premature Bergman cyclization leading to 25. Reagents and conditions: 9. DIBAL-H (1.5 equiv), CH_2_Cl_2_ (0.05 M), −78 °C, 1 h; 10. NiCl_2_ (20 mol %), CrCl_2_ (10 equiv), THF (0.005 M), 23°C, 3 h, 50% over two steps. DIBAL-H = diisobutylaluminum hydride; MS = molecular sieves.
