# Supplementary material for: Homo-Clavulyne: Evaluation of a One-Atom Ring Expansion Strategy to Stabilize the Nine-Membered Ring Enediyne Biosynthetic Precursor
Source: Synthesis (Stuttg). Author manuscript; Available in PMC 2026 Oct 1. (PMC13626215; doi:10.1055/a-2951-5568)
Supplement: Supplementary file 1 [file NIHMS2210950-supplement-1.pdf]

Supporting Information

Homo-Clavulyne: Evaluation of a One-Atom Ring Expansion Strategy to Stabilize the 9-Membered Ring Enediyne Biosynthetic Precursor

Lande Hu, Myungeun Jeong, Mohammad R. Seyedsayamdost, and Erik J. Sorensen*

Department of Chemistry, Princeton University, Princeton, New Jersey, 08544, United States

*ejs@princeton.edu

Table of Contents

Nuclear Magnetic Resonance (NMR) Spectra……S2

Three distinct strategies explored to construct fragment **6**……S22

Optimization of 1,4-borylation/oxidation……S25

Model study of Sonogashira reaction with vinyl bromide **7**……S26

Optimizations of Sonogashira fragment coupling……S27

Attempts to obtain aldehyde **21a** and **21b** via oxidation……S28

Condition screening for the ring-closing acetylide addition……S29

References……S30

Synthesis | DOI 10.1055/a-2951-5568 | © The Author(s).

Nuclear Magnetic Resonance (NMR) Spectra

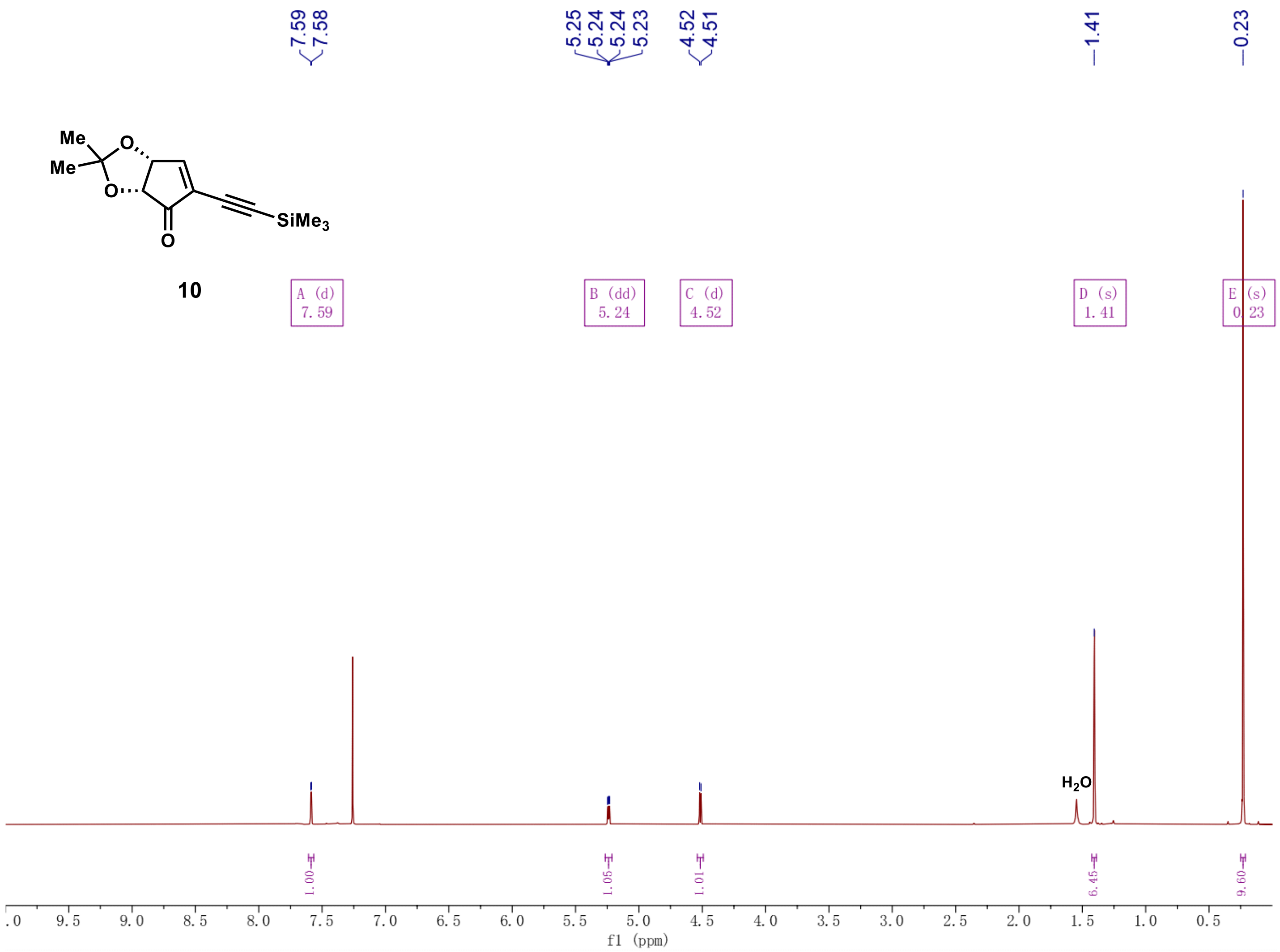

Figure S1. ^{1}H NMR spectrum of **10** in $CDCl_3$. Spectrometer frequency 500 MHz, 22 °C.

Synthesis | DOI 10.1055/a-2951-5568 | © The Author(s).

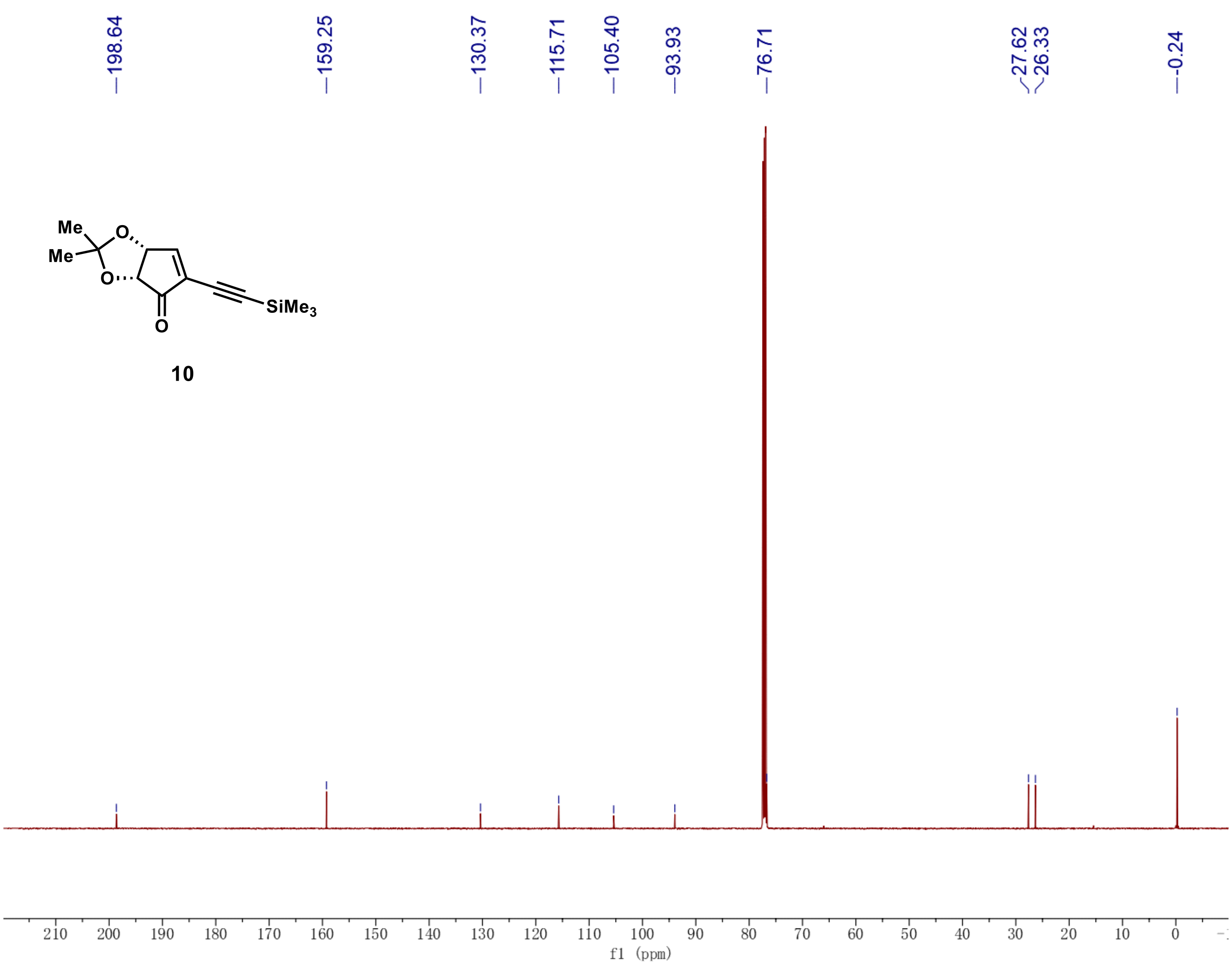

Figure S2. ^{13}C NMR spectrum of **10** in $CDCl_3$. Spectrometer frequency 126 MHz, 22 °C.

Synthesis | DOI 10.1055/a-2951-5568 | © The Author(s).

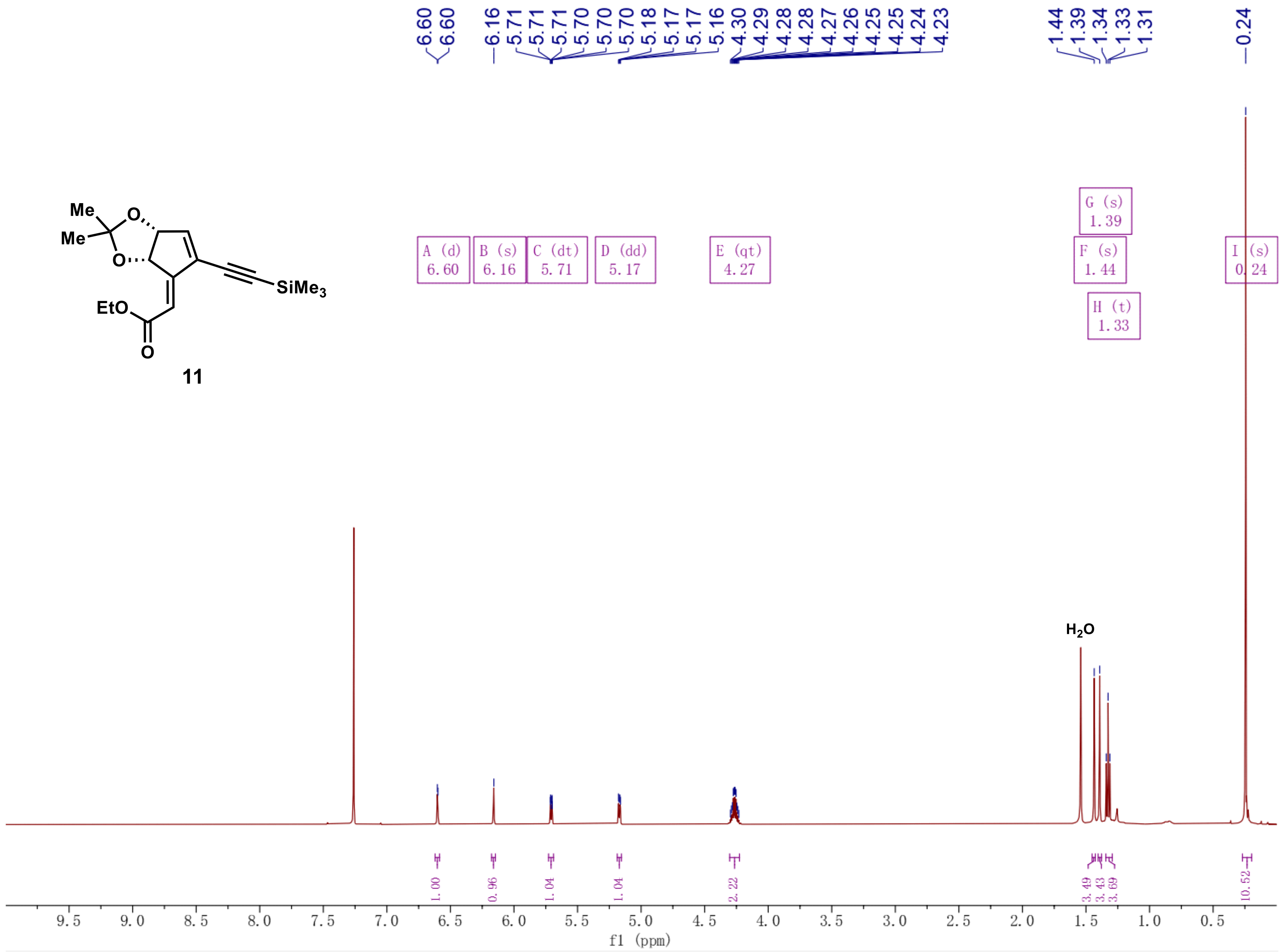

Figure S3. ^{1}H NMR spectrum of **11** in $CDCl_3$. Spectrometer frequency 500 MHz, 22 °C.

Synthesis | DOI 10.1055/a-2951-5568 | © The Author(s).

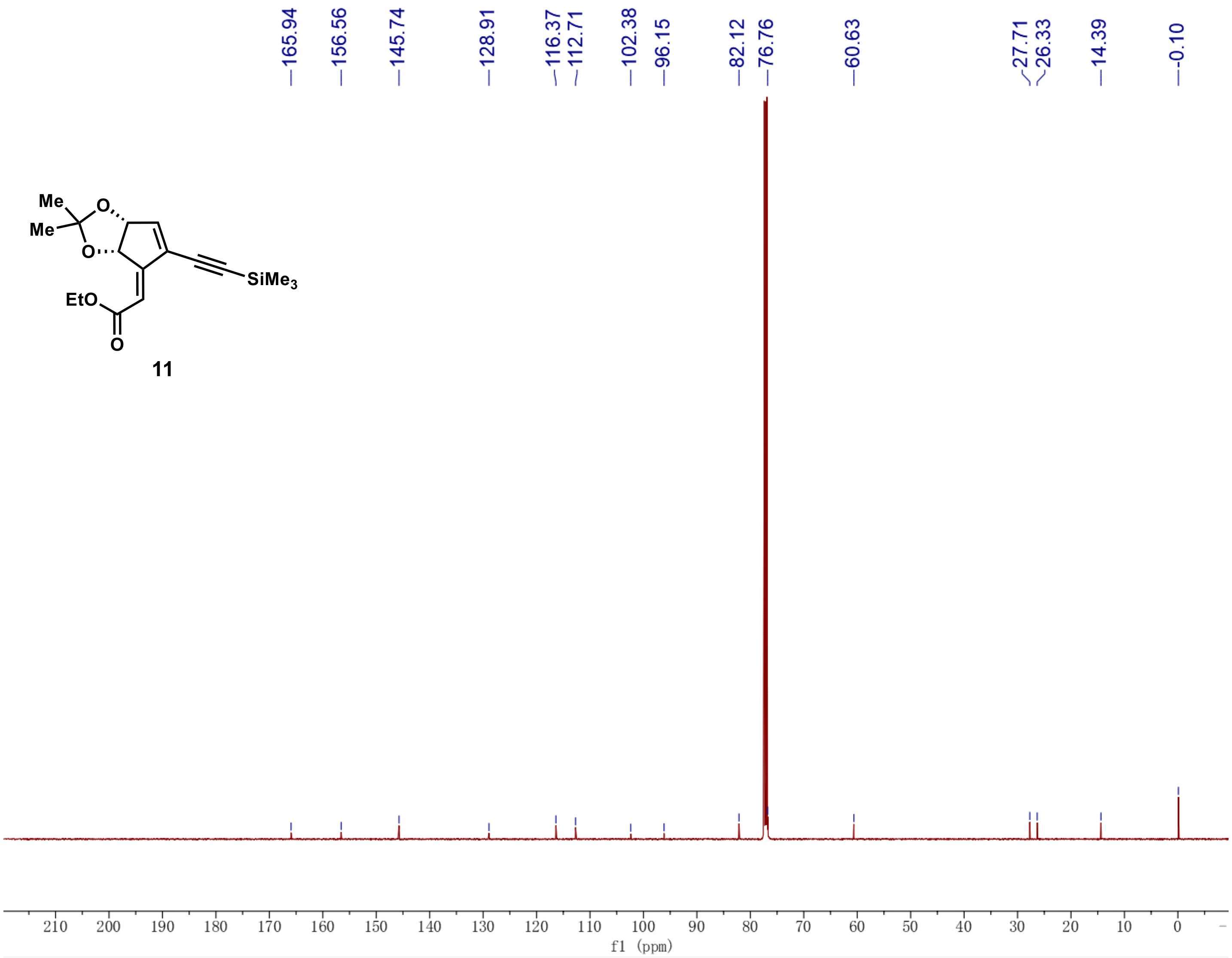

11

Figure S4. ^{13}C NMR spectrum of **11** in $CDCl_3$. Spectrometer frequency 126 MHz, 22 °C.

Synthesis | DOI 10.1055/a-2951-5568 | © The Author(s).

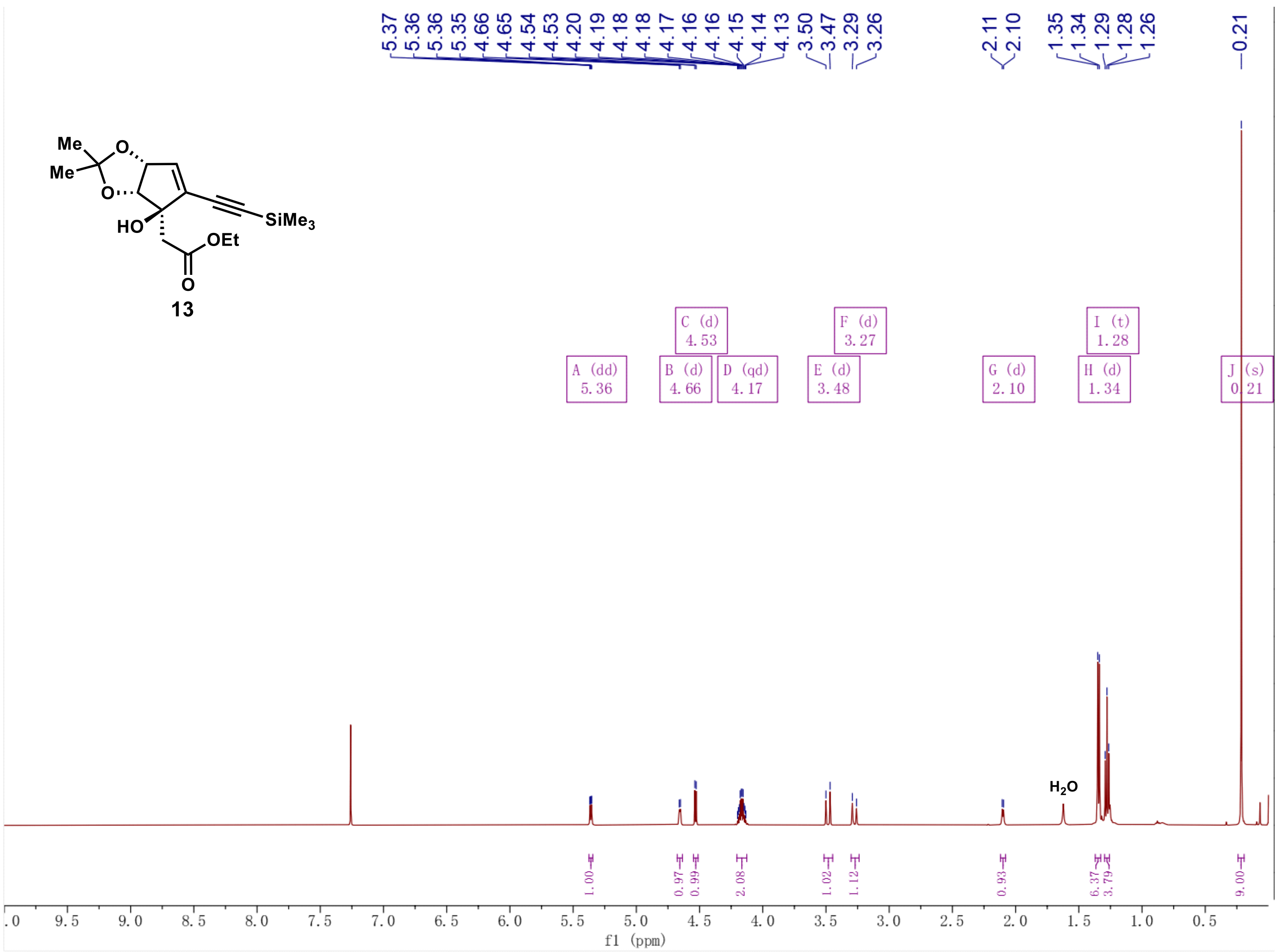

Figure S5. ^{1}H NMR spectrum of **13** in $CDCl_3$. Spectrometer frequency 500 MHz, 22 °C.

Synthesis | DOI 10.1055/a-2951-5568 | © The Author(s).

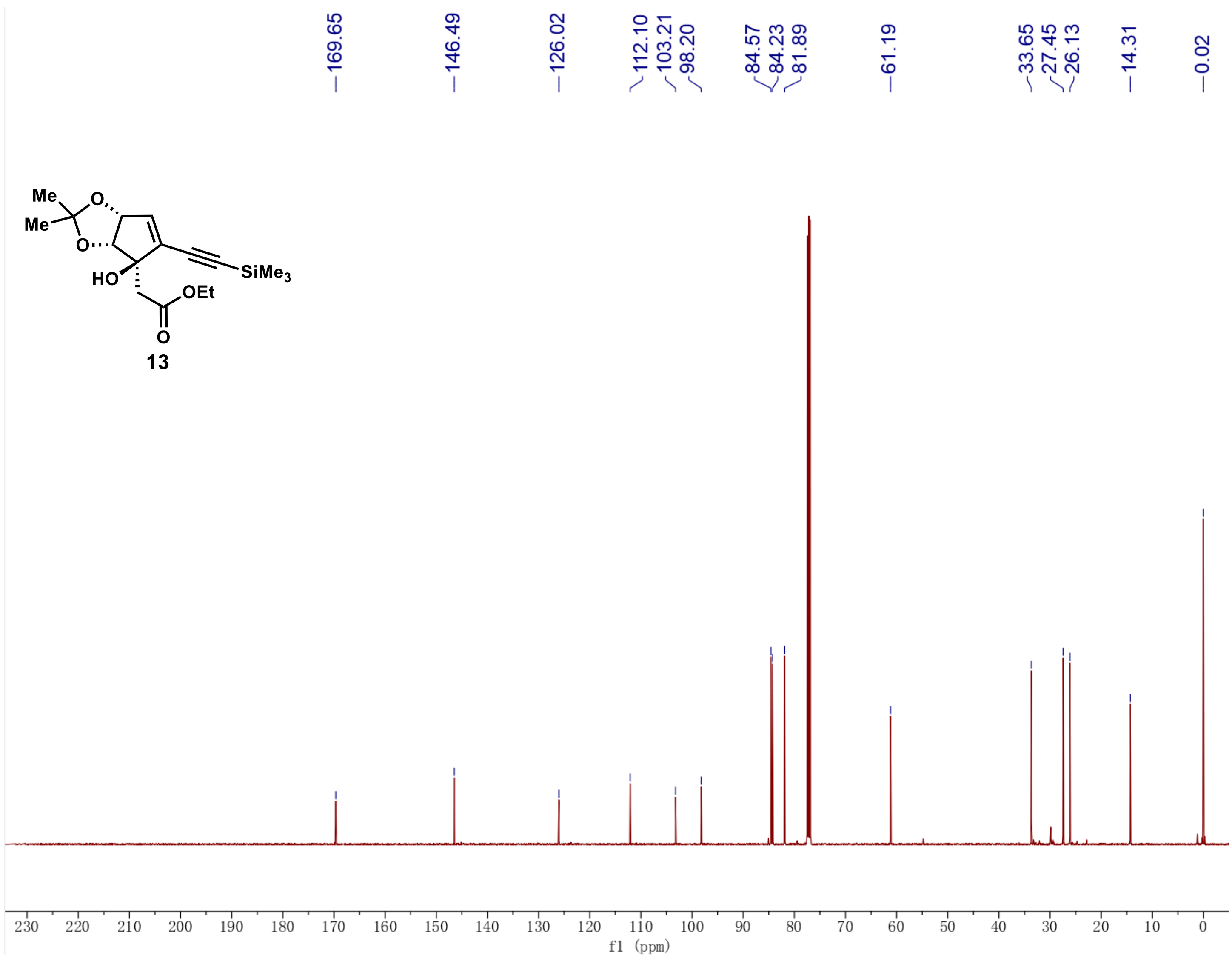

Figure S6. ^{13}C NMR spectrum of **13** in $CDCl_3$. Spectrometer frequency 126 MHz, 22 °C.

Synthesis | DOI 10.1055/a-2951-5568 | © The Author(s).

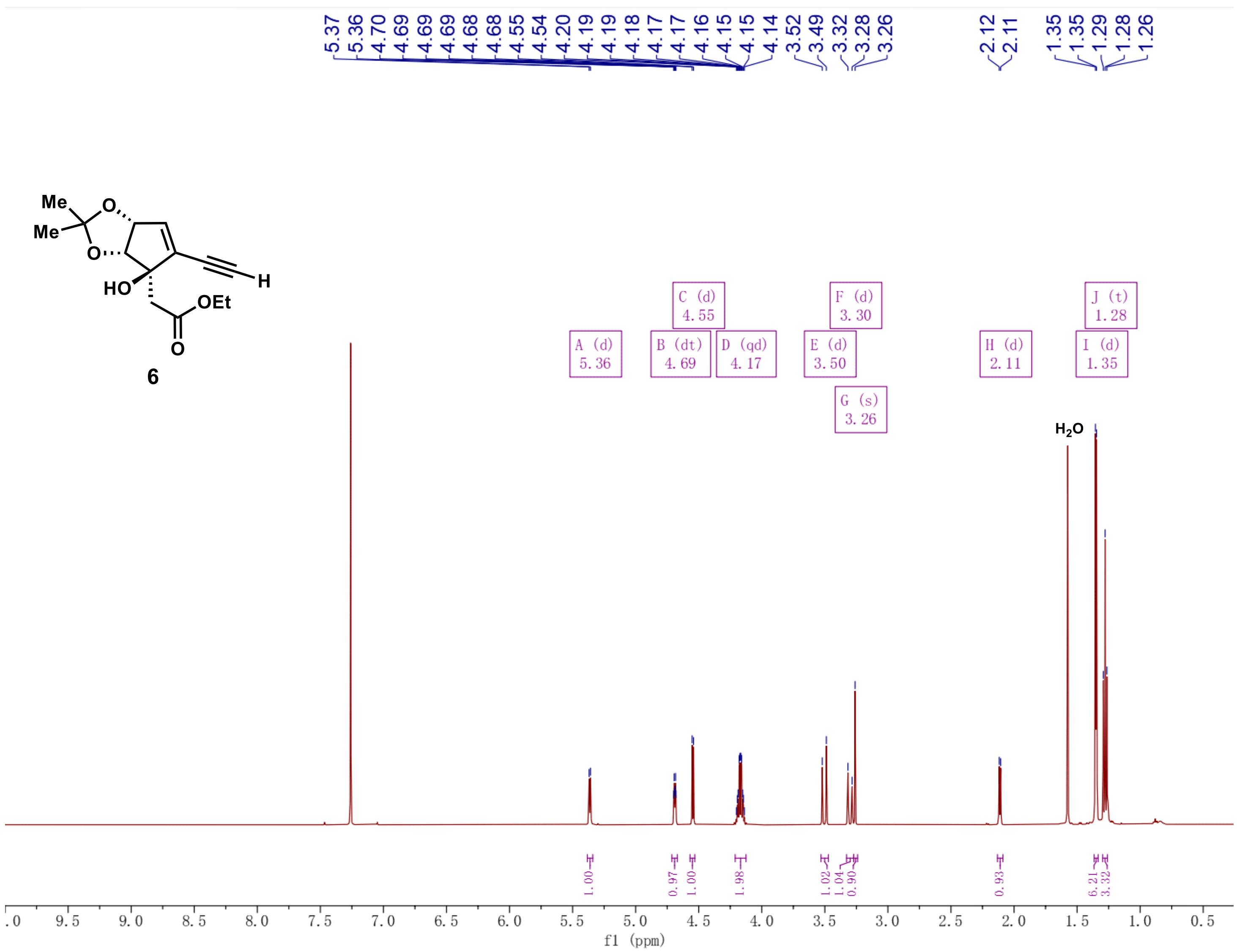

Figure S7. ^{1}H NMR spectrum of **6** in $CDCl_3$. Spectrometer frequency 500 MHz, 22 °C.

Synthesis | DOI 10.1055/a-2951-5568 | © The Author(s).

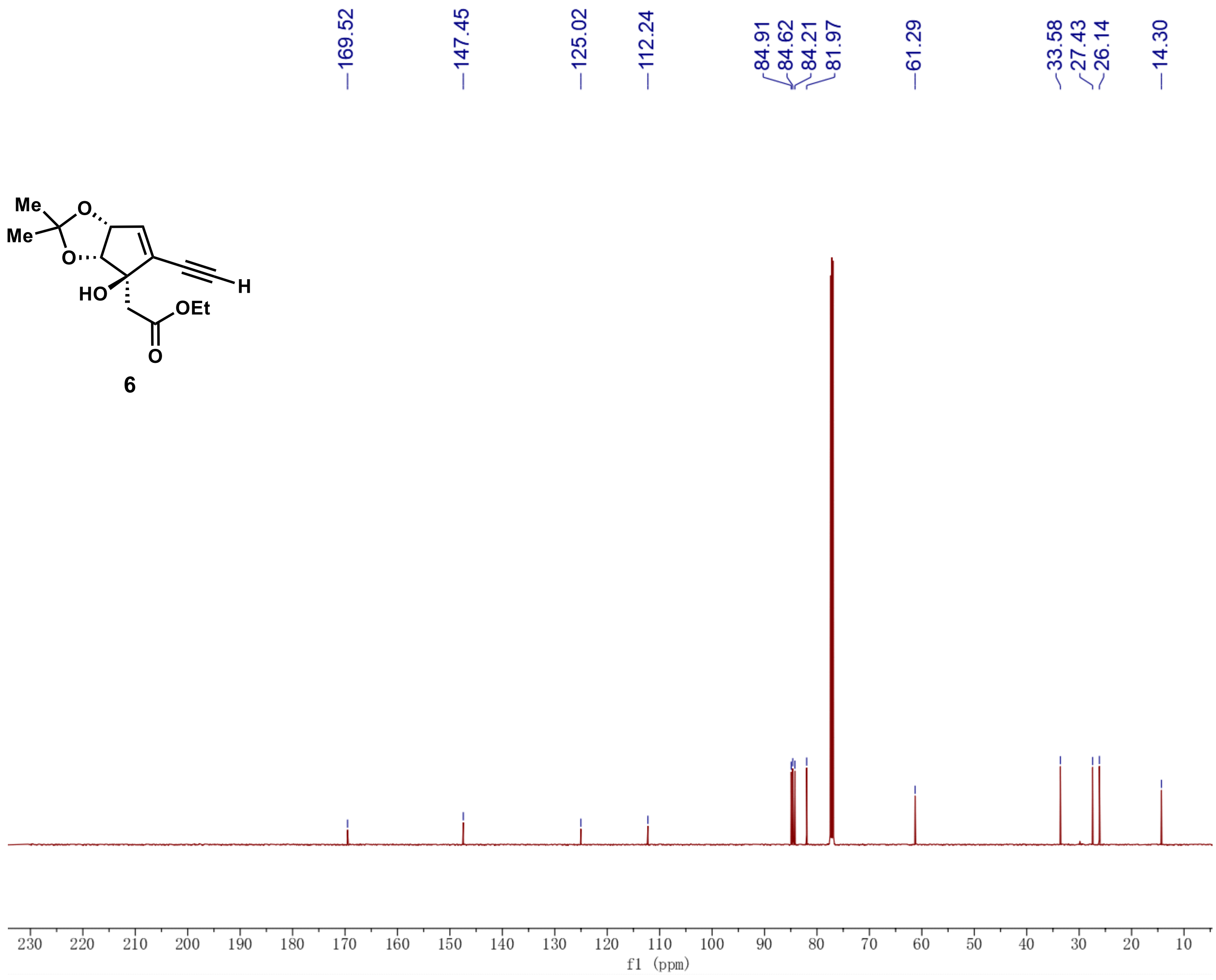

Figure S8. ^{13}C NMR spectrum of **6** in $CDCl_3$. Spectrometer frequency 126 MHz, 22 °C.

Synthesis | DOI 10.1055/a-2951-5568 | © The Author(s).

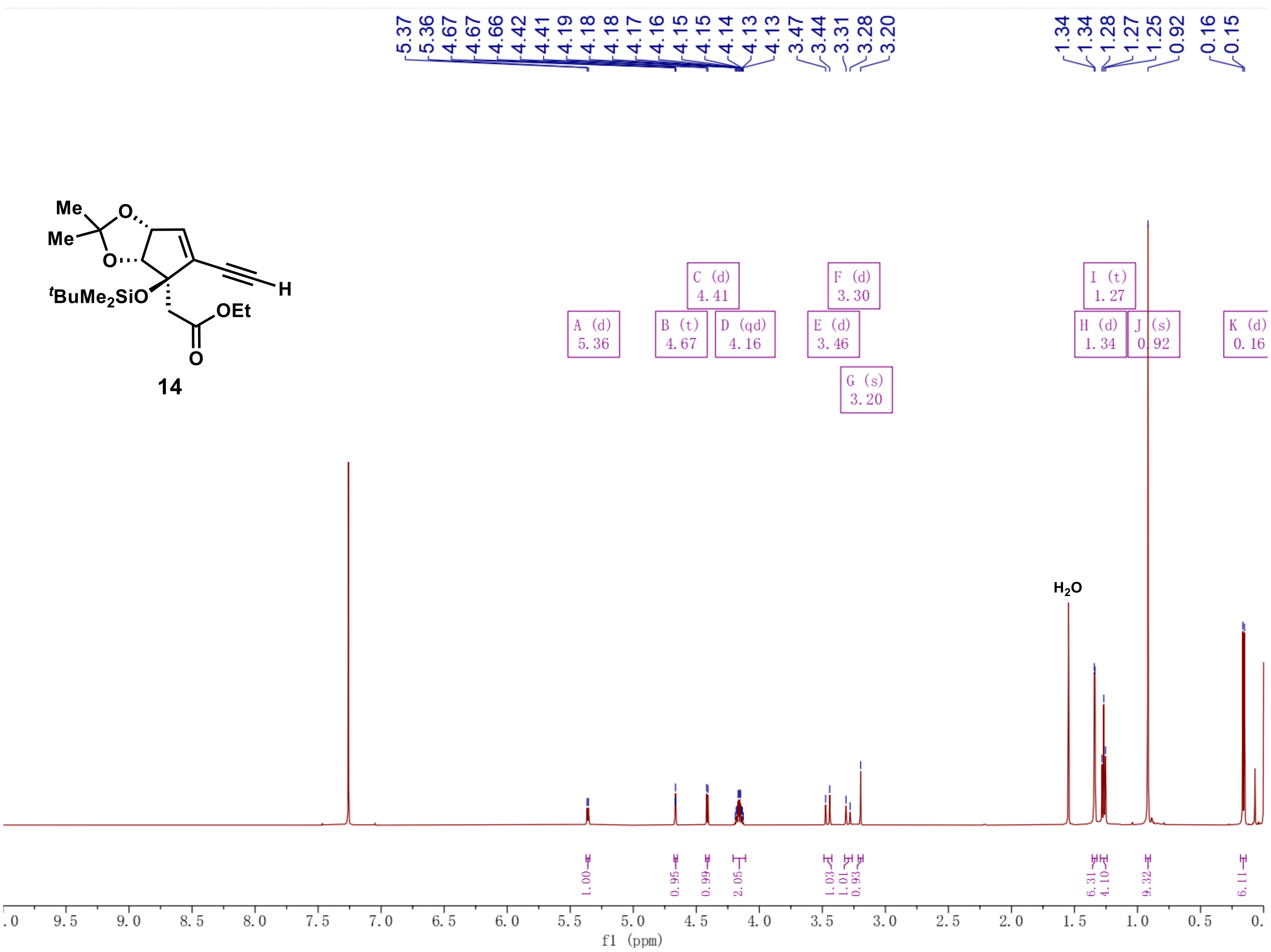

Figure S9. ^{1}H NMR spectrum of **14** in $CDCl_3$. Spectrometer frequency 500 MHz, 22 °C.

Synthesis | DOI 10.1055/a-2951-5568 | © The Author(s).

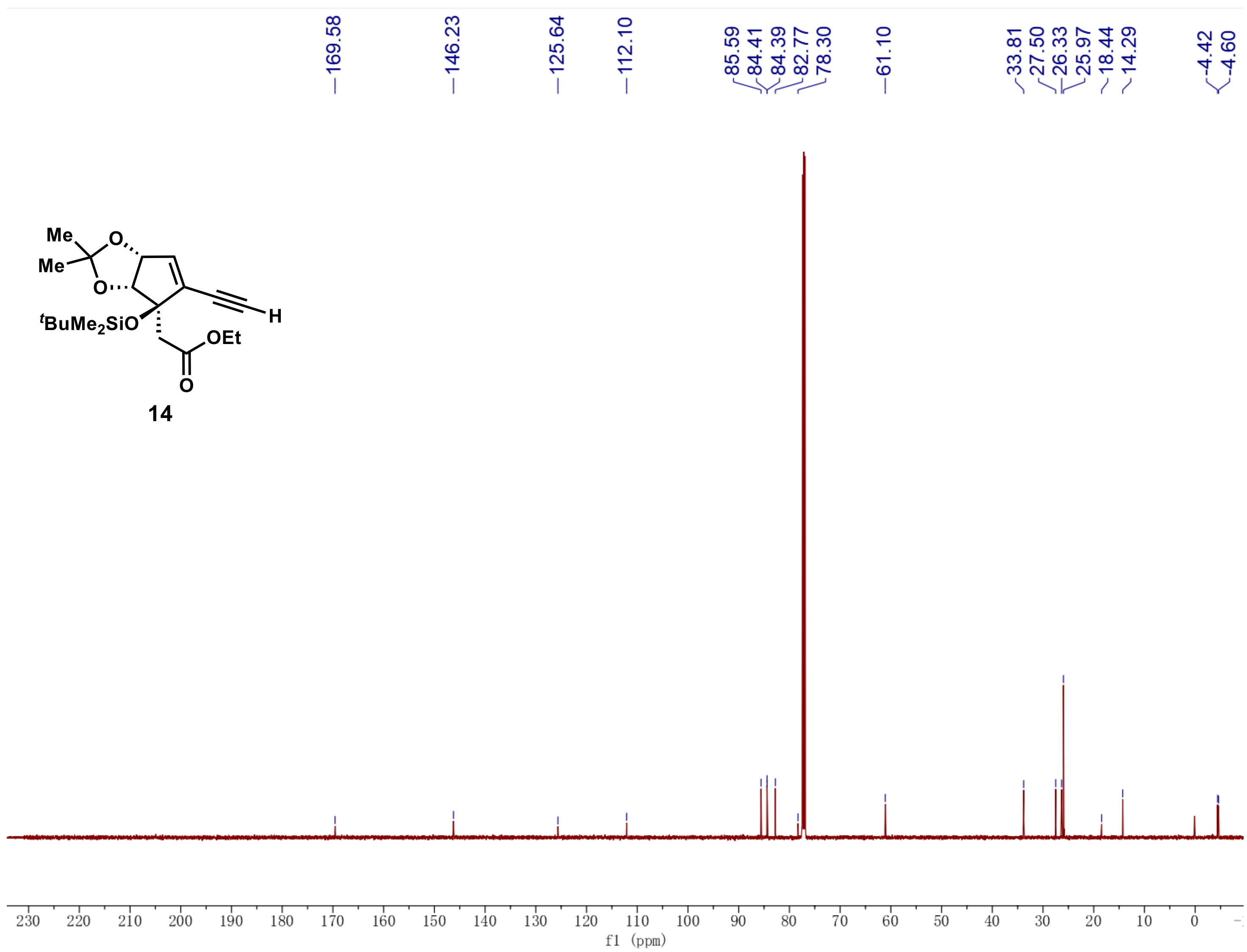

Figure S10. ^{13}C NMR spectrum of **14** in $CDCl_3$. Spectrometer frequency 126 MHz, 22 °C.

Synthesis | DOI 10.1055/a-2951-5568 | © The Author(s).

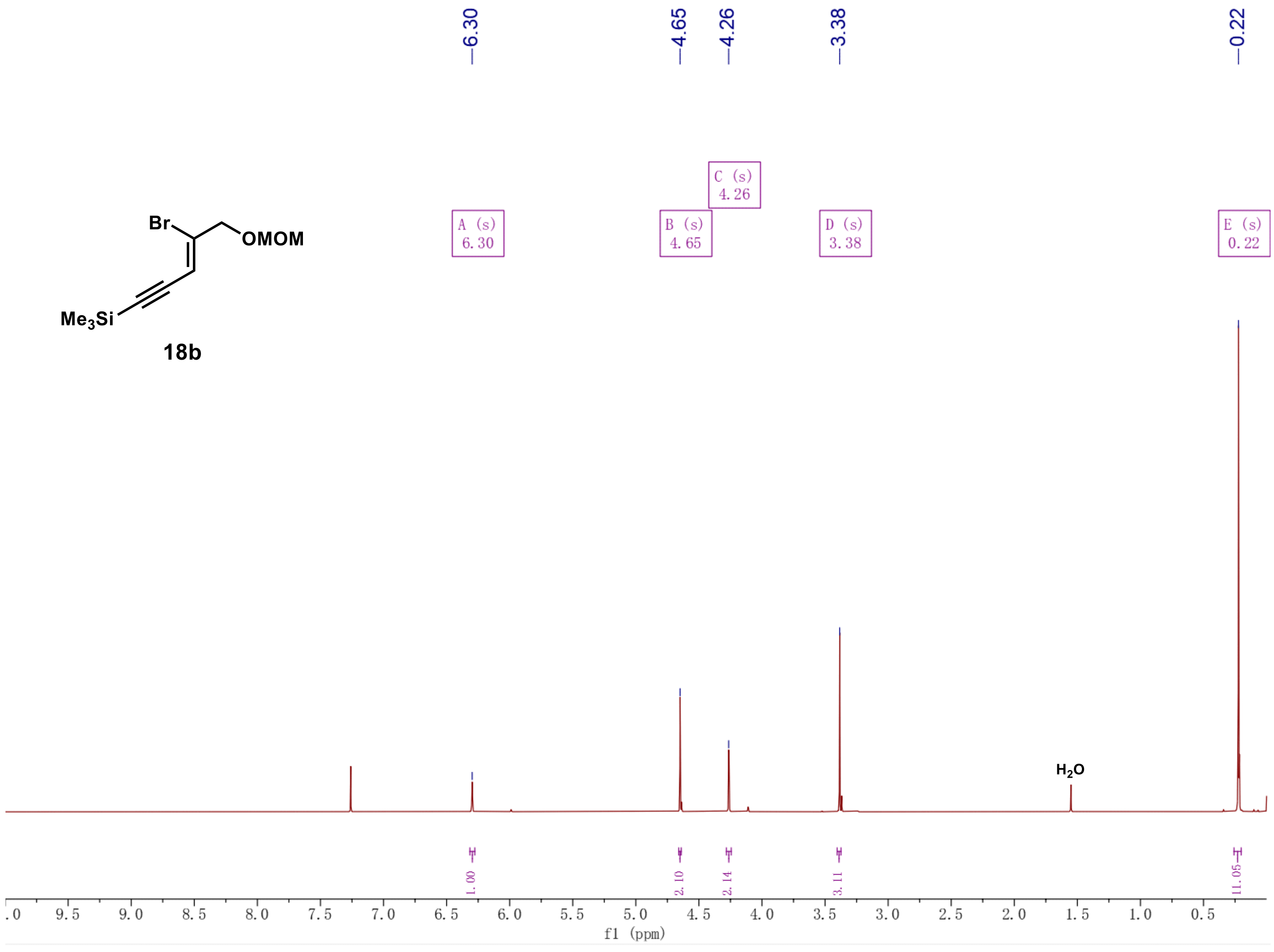

Figure S11. ^{1}H NMR spectrum of **18b** in $CDCl_3$. Spectrometer frequency 500 MHz, 22 °C.

Synthesis | DOI 10.1055/a-2951-5568 | © The Author(s).

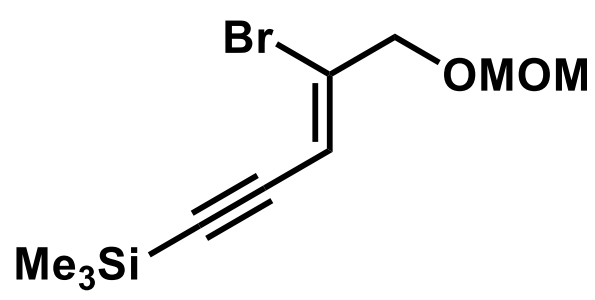

18b

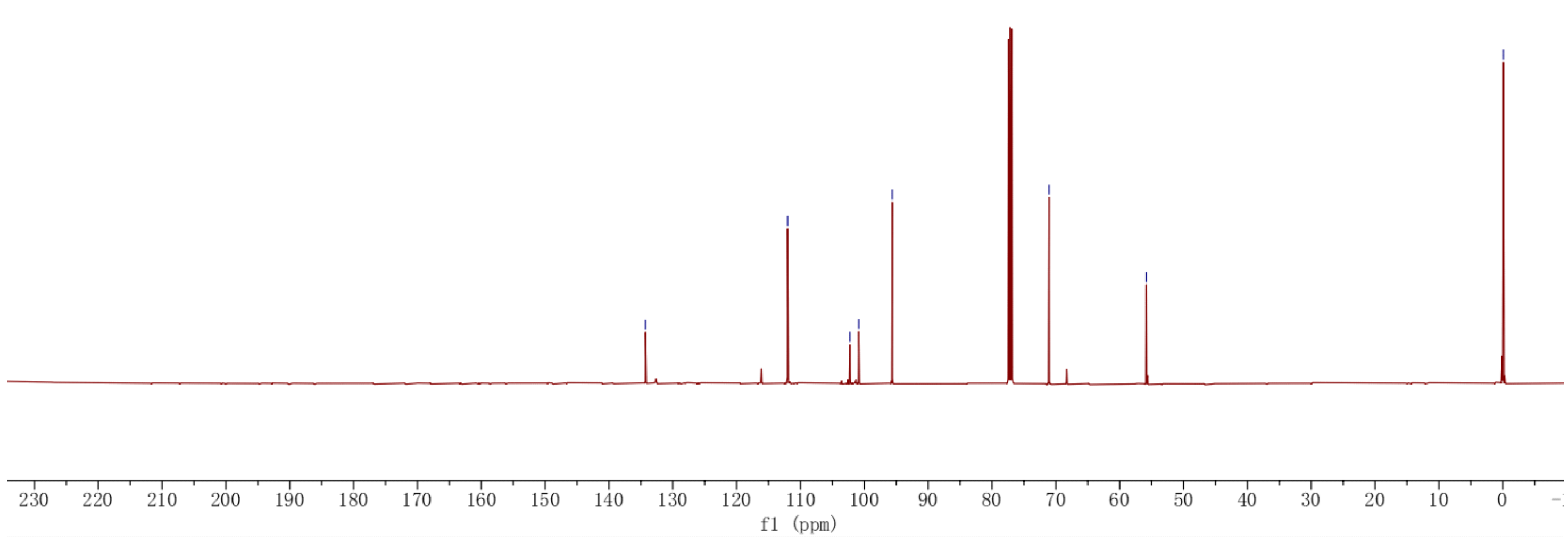

Figure S12. ^{13}C NMR spectrum of **18b** in $CDCl_3$. Spectrometer frequency 126 MHz, 22 °C.

Synthesis | DOI 10.1055/a-2951-5568 | © The Author(s).

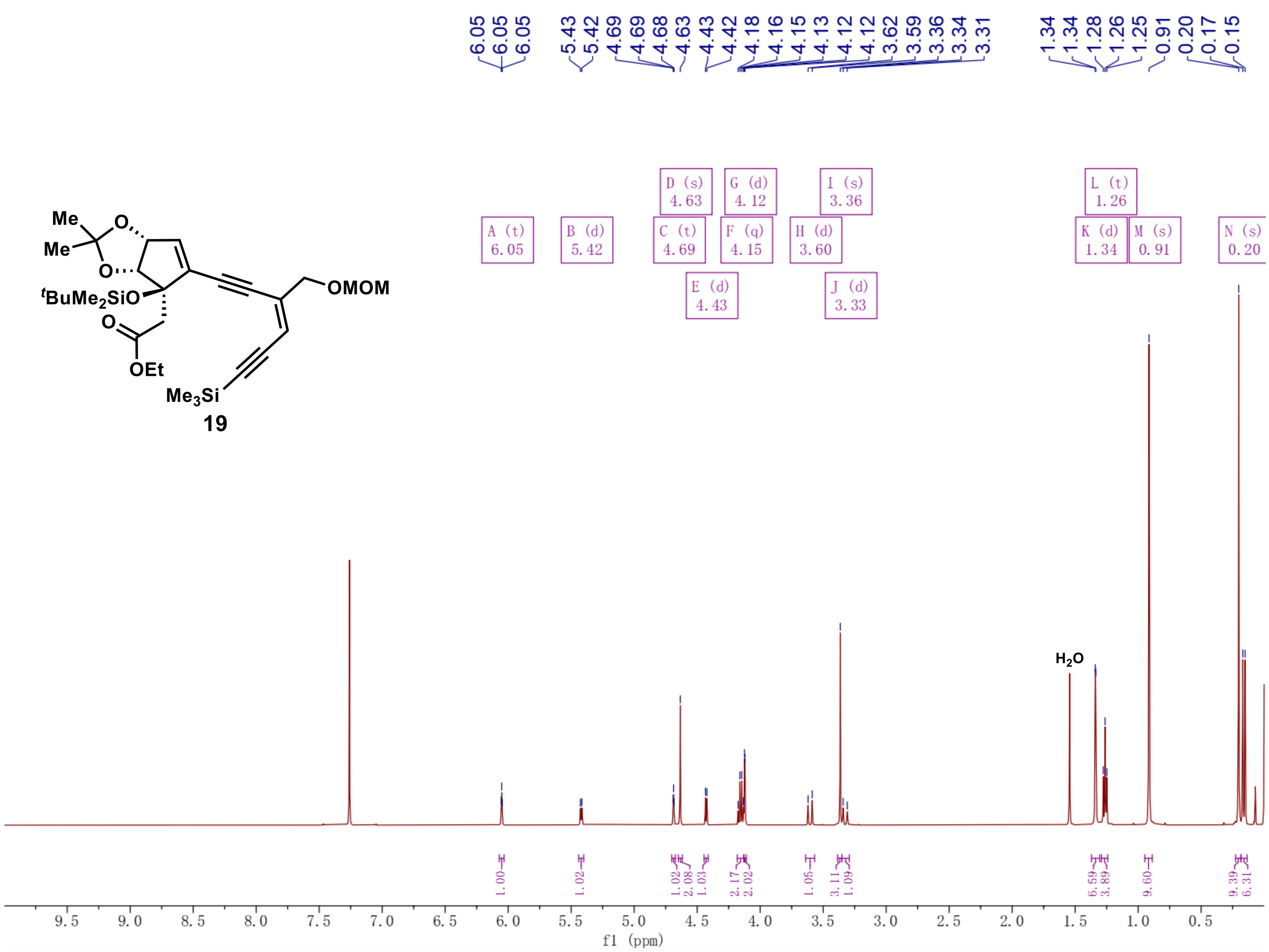

Figure S13. ^{1}H NMR spectrum of **19** in $CDCl_3$. Spectrometer frequency 500 MHz, 22 °C.

Synthesis | DOI 10.1055/a-2951-5568 | © The Author(s).

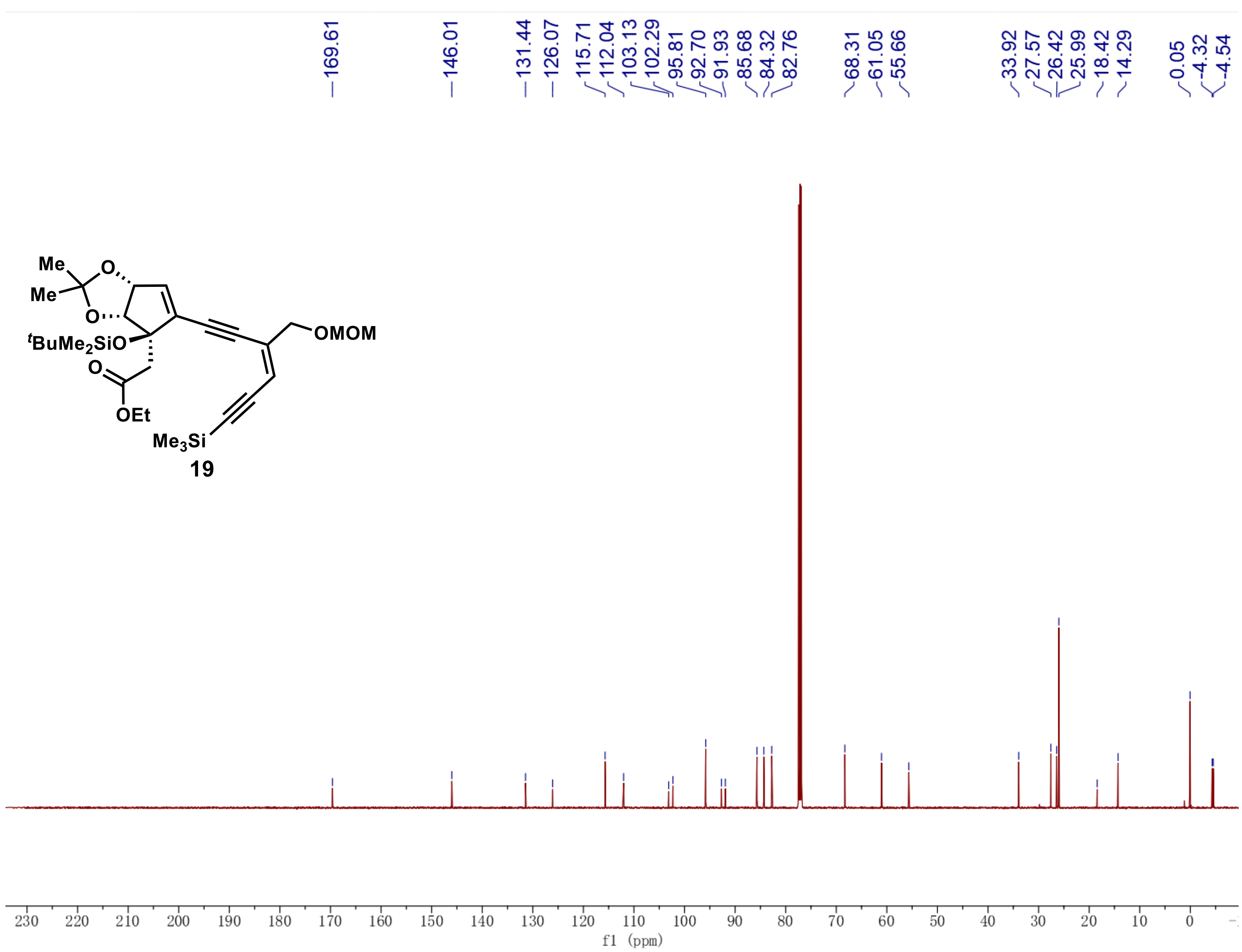

Figure S14. ^{13}C NMR spectrum of **19** in $CDCl_3$. Spectrometer frequency 126 MHz, 22 °C.

Synthesis | DOI 10.1055/a-2951-5568 | © The Author(s).

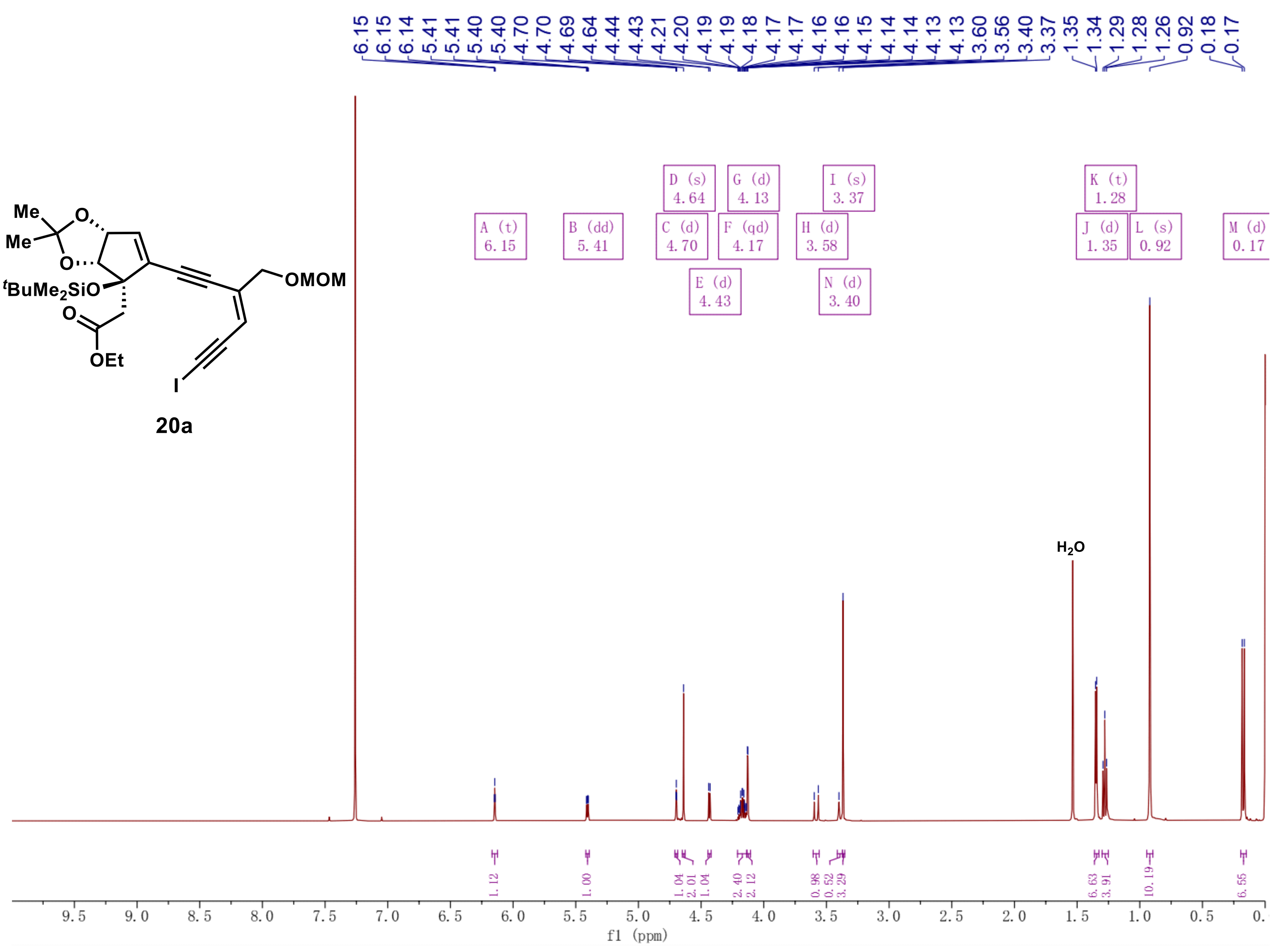

Figure S15. ^{1}H NMR spectrum of **20a** in $CDCl_3$. Spectrometer frequency 500 MHz, 22 °C.

Synthesis | DOI 10.1055/a-2951-5568 | © The Author(s).

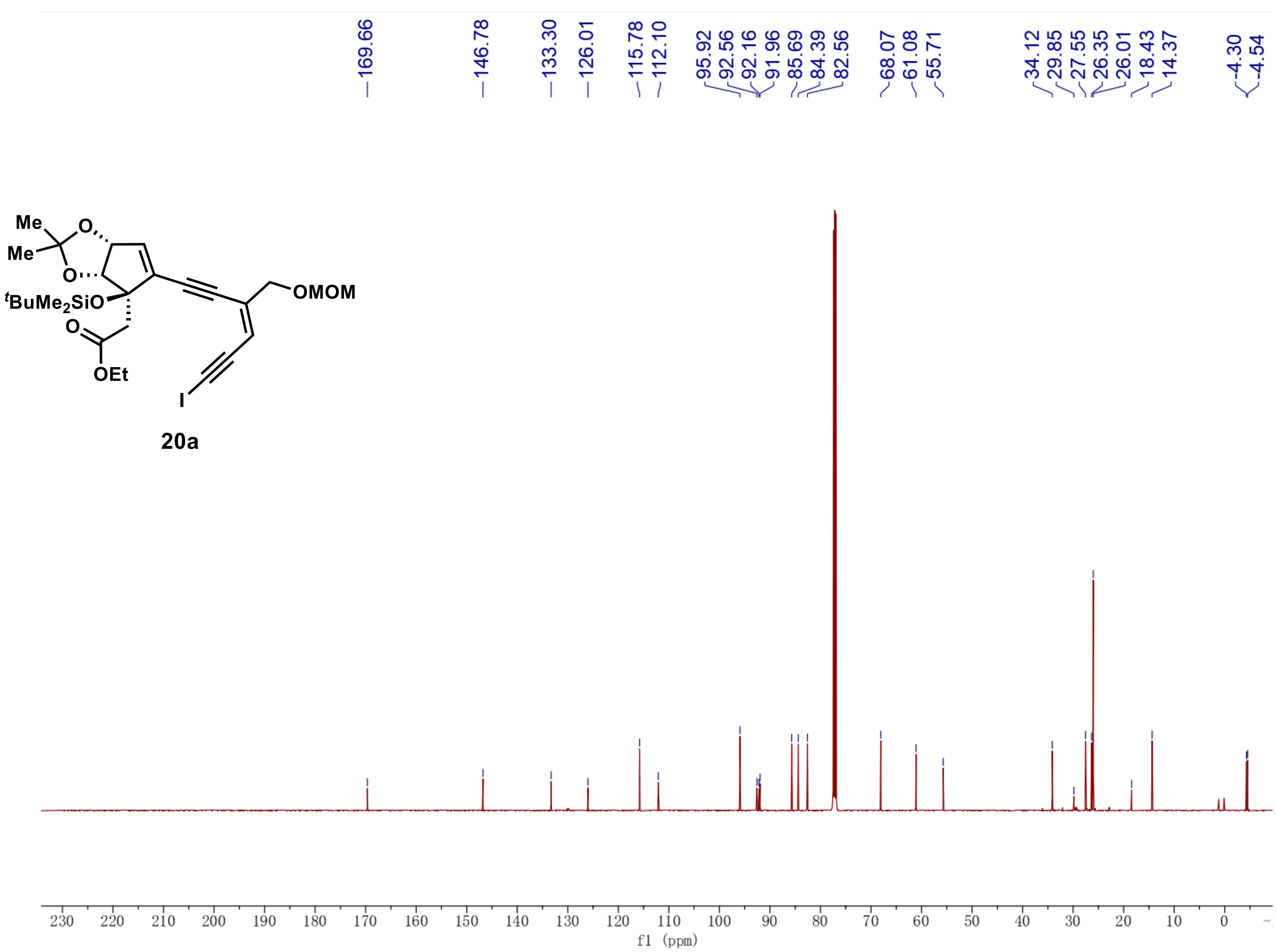

20a

Figure S16. ^{13}C NMR spectrum of **20a** in $CDCl_3$. Spectrometer frequency 126 MHz, 22 °C.

Synthesis | DOI 10.1055/a-2951-5568 | © The Author(s).

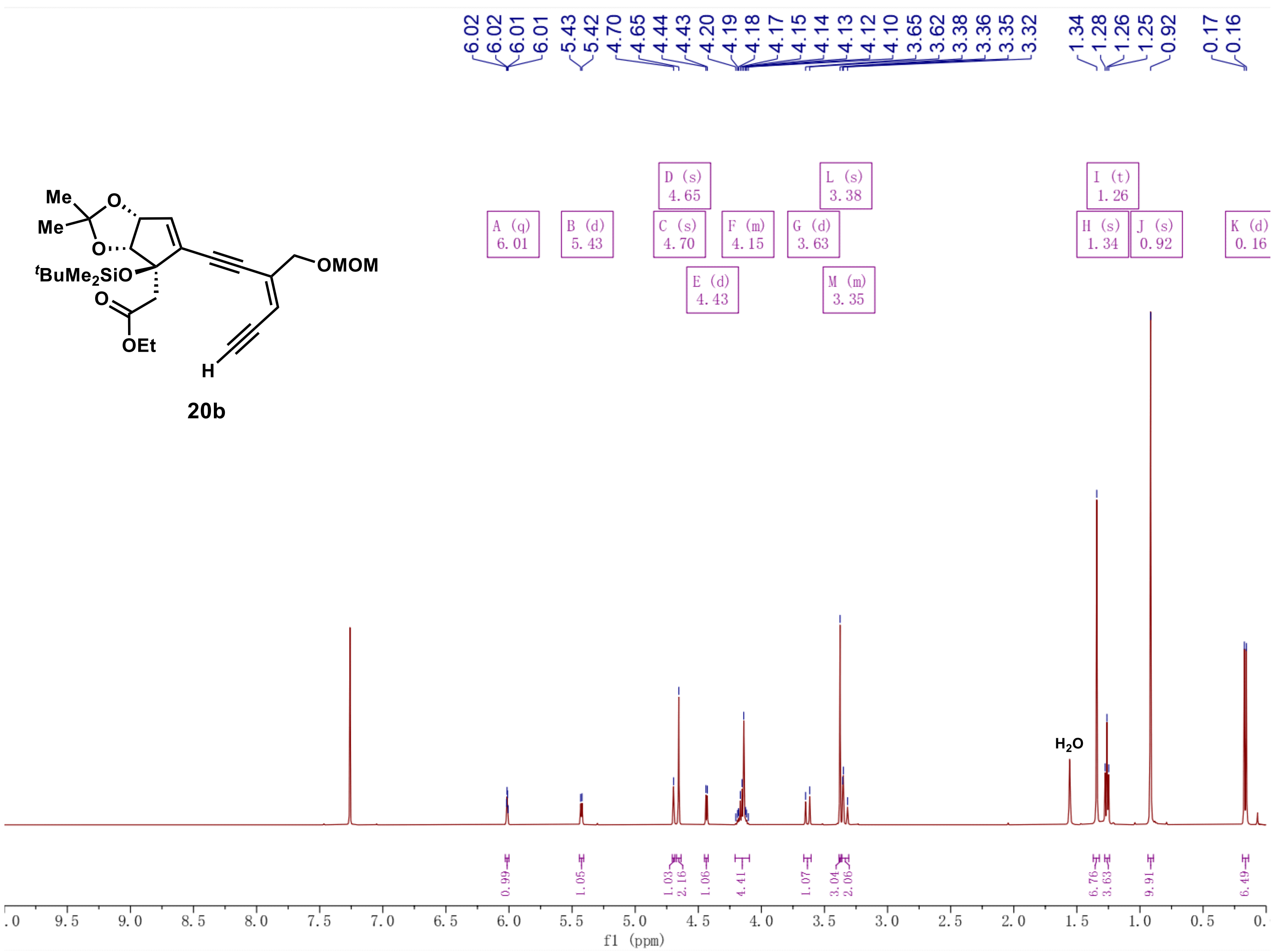

Figure S17. ¹H NMR spectrum of **20b** in $CDCl_3$. Spectrometer frequency 500 MHz, 22 °C.

Synthesis | DOI 10.1055/a-2951-5568 | © The Author(s).

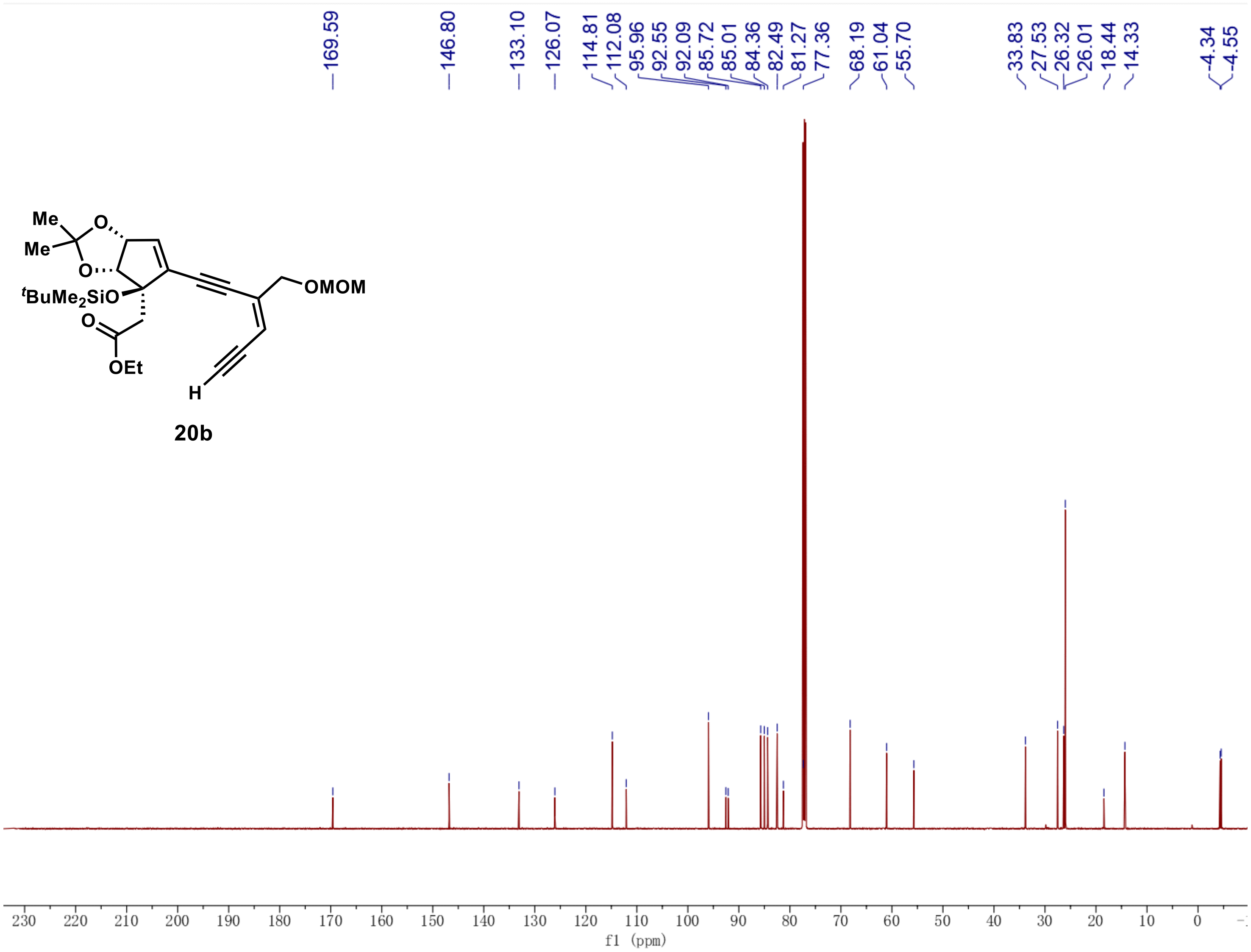

Figure S18. ^{13}C NMR spectrum of **20b** in $CDCl_3$. Spectrometer frequency 126 MHz, 22 °C.

Synthesis | DOI 10.1055/a-2951-5568 | © The Author(s).

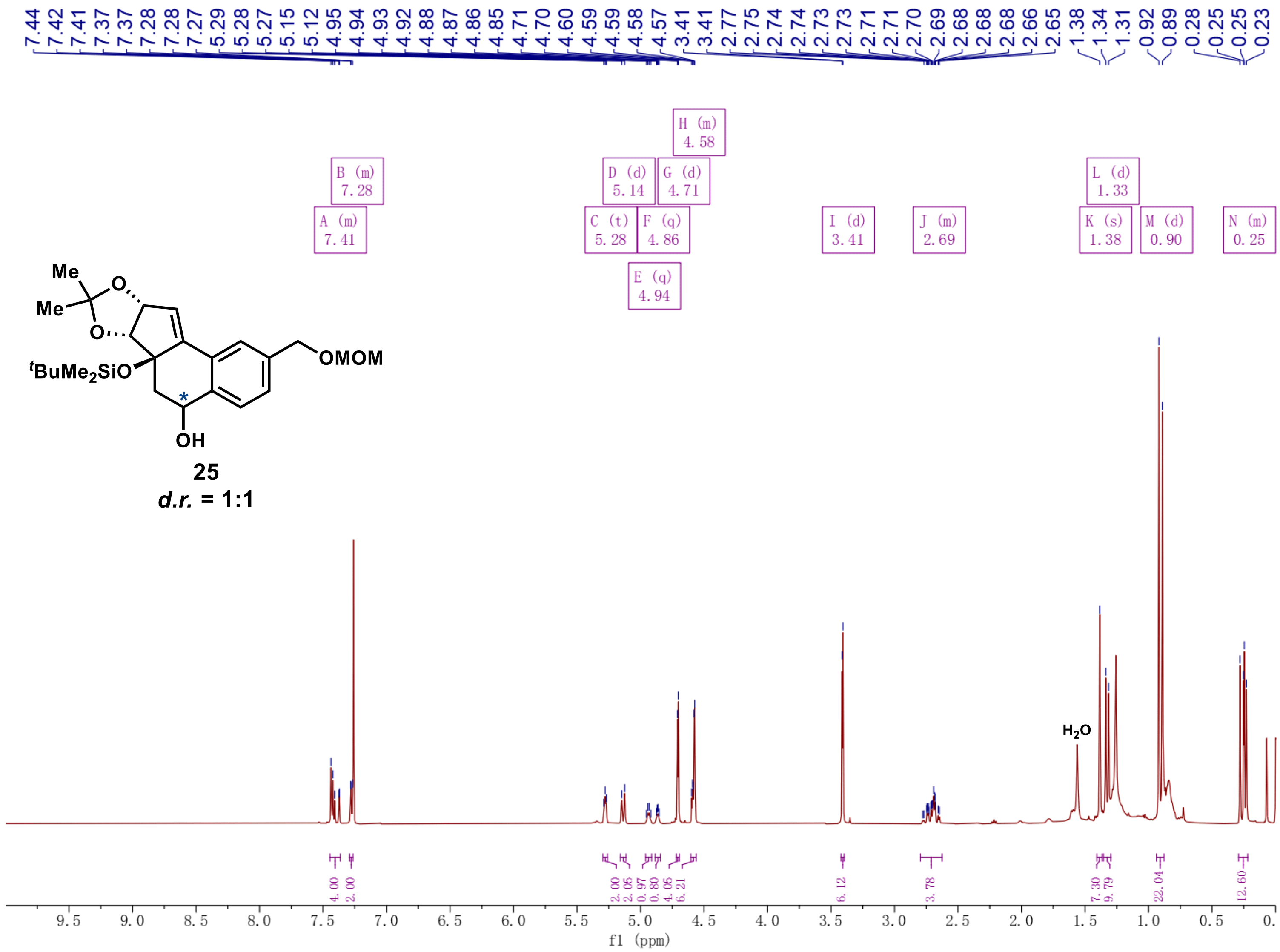

Figure S19. ^{1}H NMR spectrum of **25** in $CDCl_3$. Spectrometer frequency 500 MHz, 22 °C.

Synthesis | DOI 10.1055/a-2951-5568 | © The Author(s).

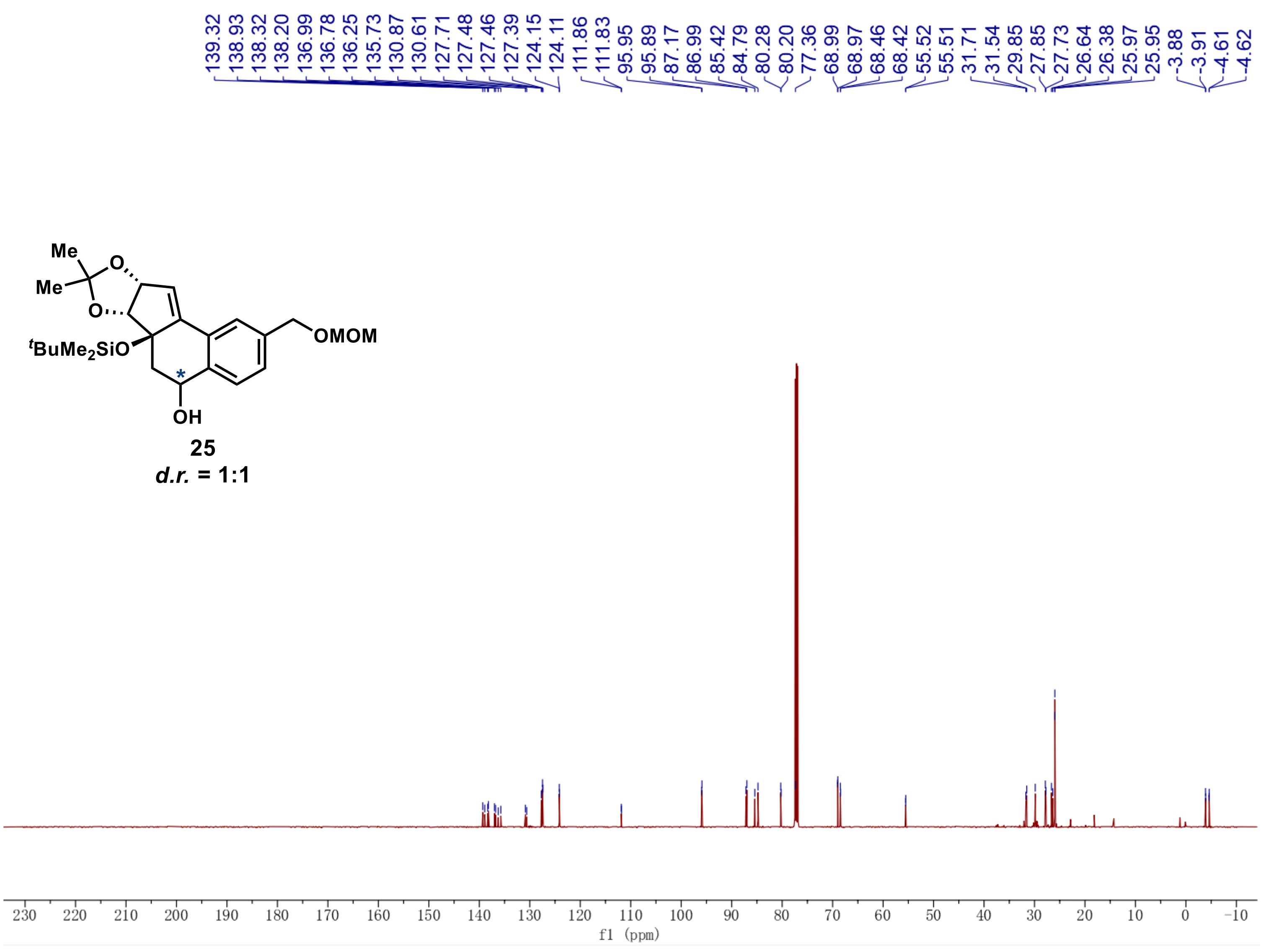

Figure S20. ^{13}C NMR spectrum of **25** in $CDCl_3$. Spectrometer frequency 126 MHz, 22 °C.

Synthesis | DOI 10.1055/a-2951-5568 | © The Author(s).

Three distinct strategies explored to construct fragment 6

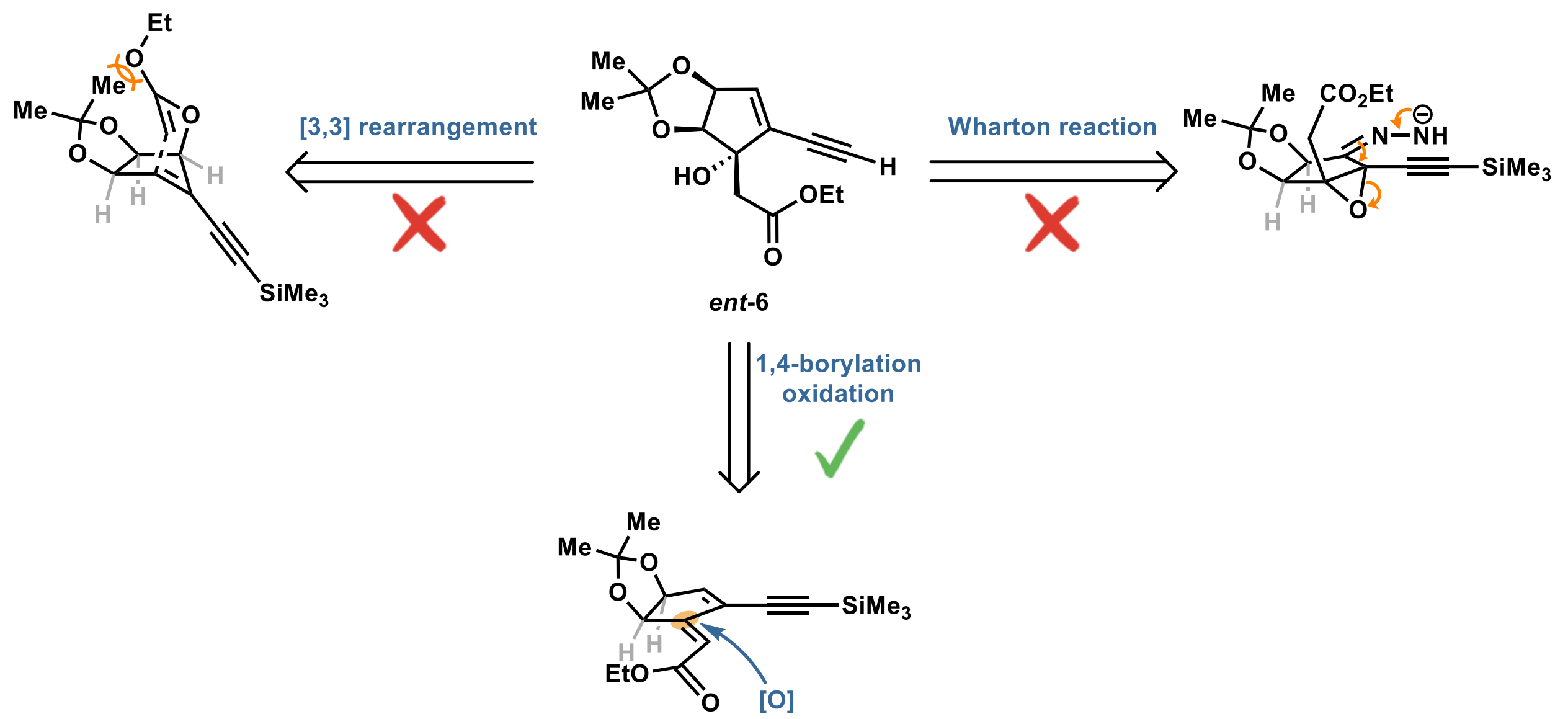

Scheme S1. Three distinct pathways for fragment **6**. The [3,3] rearrangement strategy was abandoned due to low material throughput and scalability. The Wharton reaction strategy was abandoned due to functional group tolerance issues. The 1,4-borylation/oxidation strategy was described in the main text.

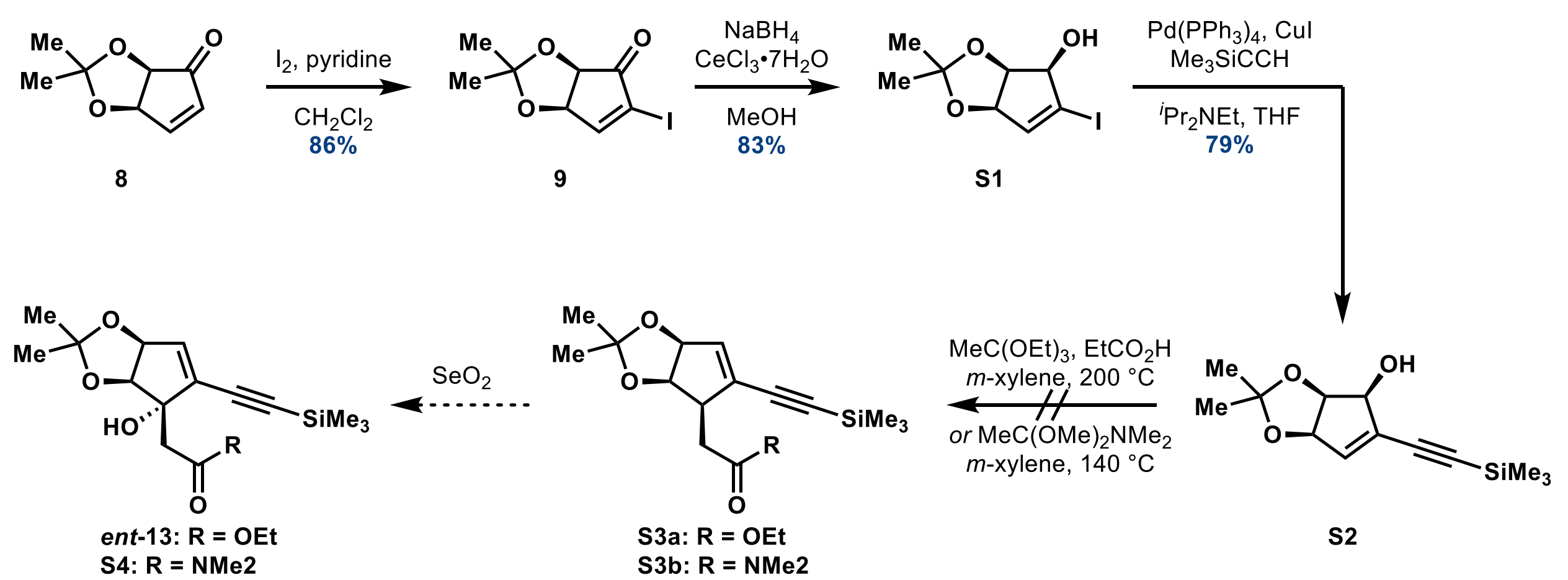

Scheme S2. Synthesis of **S2** from **8** and the failed attempts of Johnson– and Eschenmoser–Claisen rearrangements.

Synthesis | DOI 10.1055/a-2951-5568 | © The Author(s).

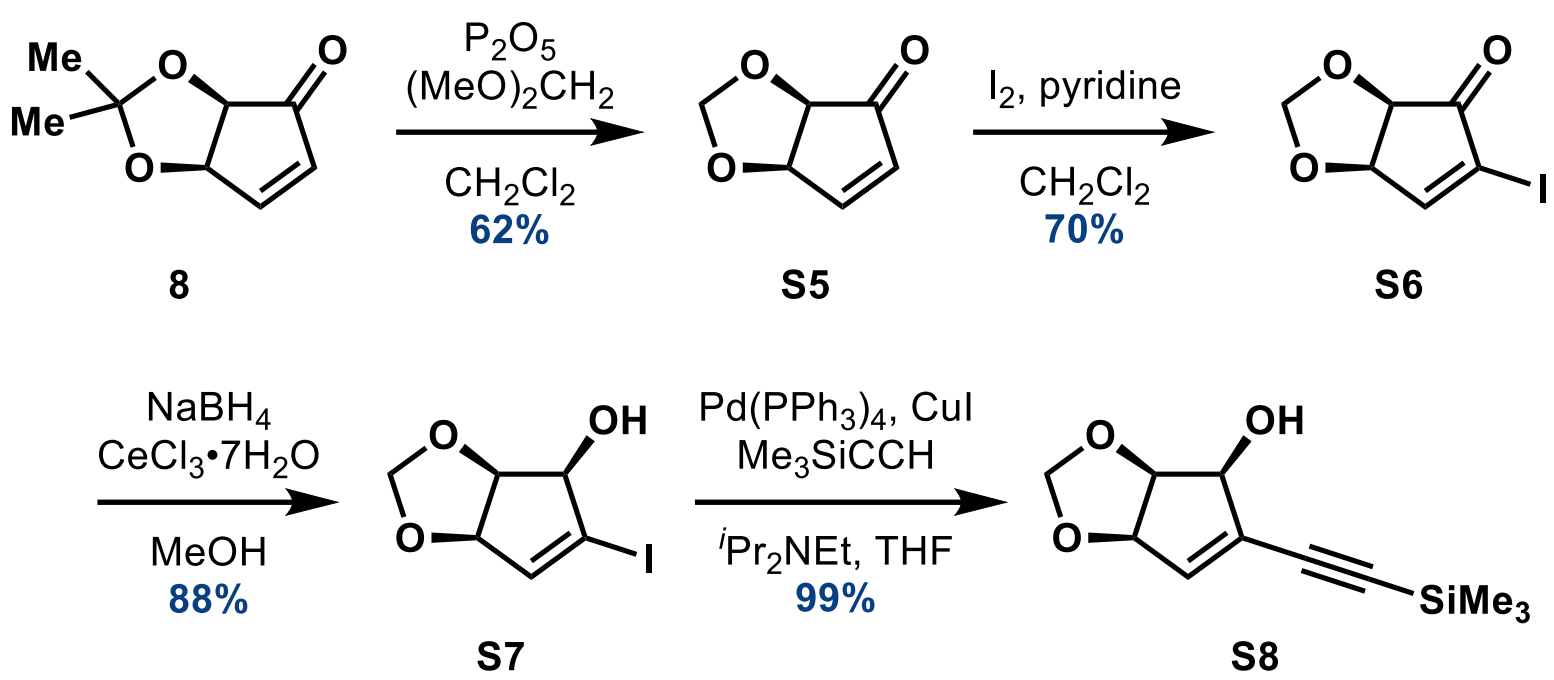

Scheme S3. Synthesis of **S7** and **S8** from **8** in preparation for the [3,3] rearrangement with less sterically hindering methylene acetal instead of acetonide.

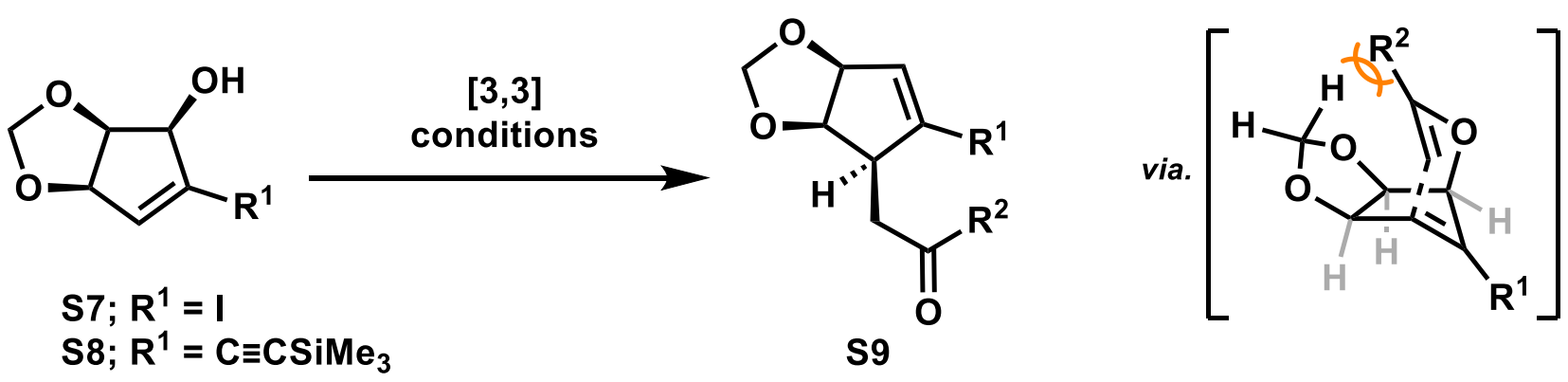

[3,3] conditions

R groups	conditions	results
R^1 = I, R^2 = NMe_2	$MeC(OMe)_2NMe_2$, *m*-Xylene, 180 °C, 24 h<br>20 mg scale	46%[a]
R^1 = I, R^2 = OEt	$MeC(OEt)_3$, $EtCO_2H$, *m*-Xylene, 180 °C, 24 h<br>20 mg scale	decomposition
R^1 = C≡CSiMe$_3$, R^2 = NMe_2	$MeC(OMe)_2NMe_2$, *m*-Xylene, 180 °C, 24 h<br>20 mg scale	decomposition
R^1 = C≡CSiMe$_3$, R^2 = OEt	$MeC(OEt)_3$, $EtCO_2H$, *m*-Xylene, 180°C, 60 h<br>20 mg scale	60%[a]
R^1 = C≡CSiMe$_3$, R^2 = H	$Hg(OAc)_2$, NEt_3, ^{n}butyl vinyl ether, 140 °C, 24 h<br>20 mg scale	decomposition
R^1 = C≡CSiMe$_3$, R^2 = OEt	$MeC(OEt)_3$, $EtCO_2H$, *m*-Xylene, 200 °C, 36 h<br>20 mg scale	72%[a]
R^1 = C≡CSiMe$_3$, R^2 = OEt	$MeC(OEt)_3$, $EtCO_2H$, 200 °C, 36 h<br>20 mg scale	decomposition
R^1 = C≡CSiMe$_3$, R^2 = OEt	$MeC(OEt)_3$, $EtCO_2H$, *m*-Xylene, 200 °C, 36 h<br>1 g scale	S9: 11%[b]<br>S8: 81%[b]
R^1 = C≡CSiMe$_3$, R^2 = OEt	$MeC(OEt)_3$, $EtCO_2H$, *m*-Xylene, 200 °C, 17 days<br>600 mg scale	S9: 23%[b]

Table S1. Attempts for [3,3] rearrangements of **S7** and **S8**. [a] ^1H NMR yields with 1,2,4,5-tetrachlorobenzene as internal standard. [b] isolated yields.

Synthesis | DOI 10.1055/a-2951-5568 | © The Author(s).

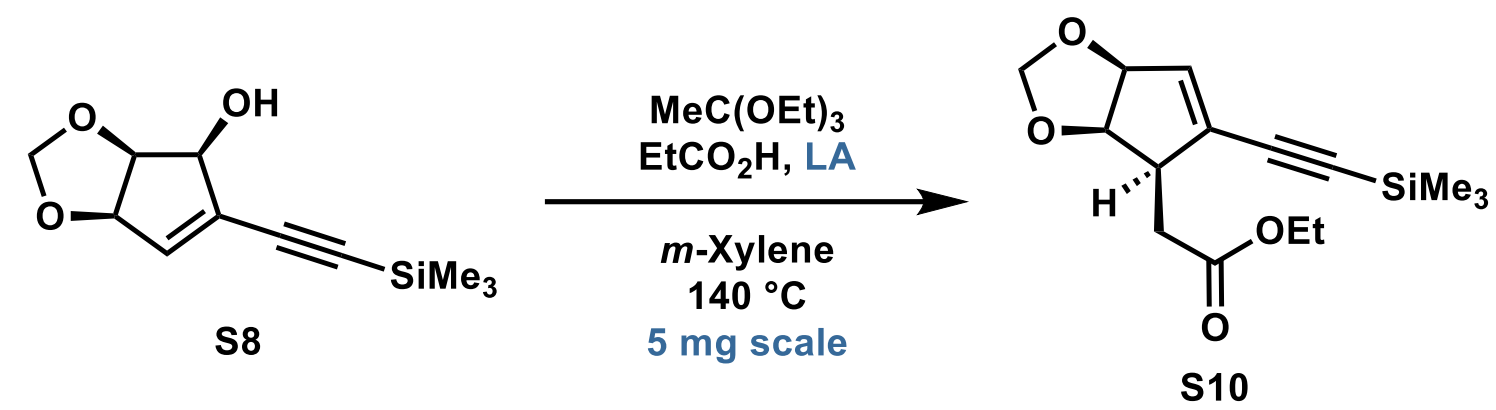

[3,3] conditions

Lewis acid	results	Lewis acid	results
$Al^{i}Bu_3$	no reaction	$Bi(OTf)_3$	decomposition
$AlCl_3$	no reaction	$In(OTf)_3$	decomposition
$AlClEt_2$	no reaction	$SnCl_4$	decomposition
$Sc(OTf)_3$	decomposition	$TiCl_4$	no reaction
$Zn(OTf)_2$	decomposition	$Hg(OAc)_2$	no reaction
$Cu(OTf)_2$	decomposition	$Pd(OAc)_2$	decomposition
$Yb(OTf)_3$	decomposition	$B(C_6F_5)$	no reaction

Table S2. Attempts for Lewis-acid-catalyzed [3,3] rearrangements of **S8**. LA = Lewis acid.

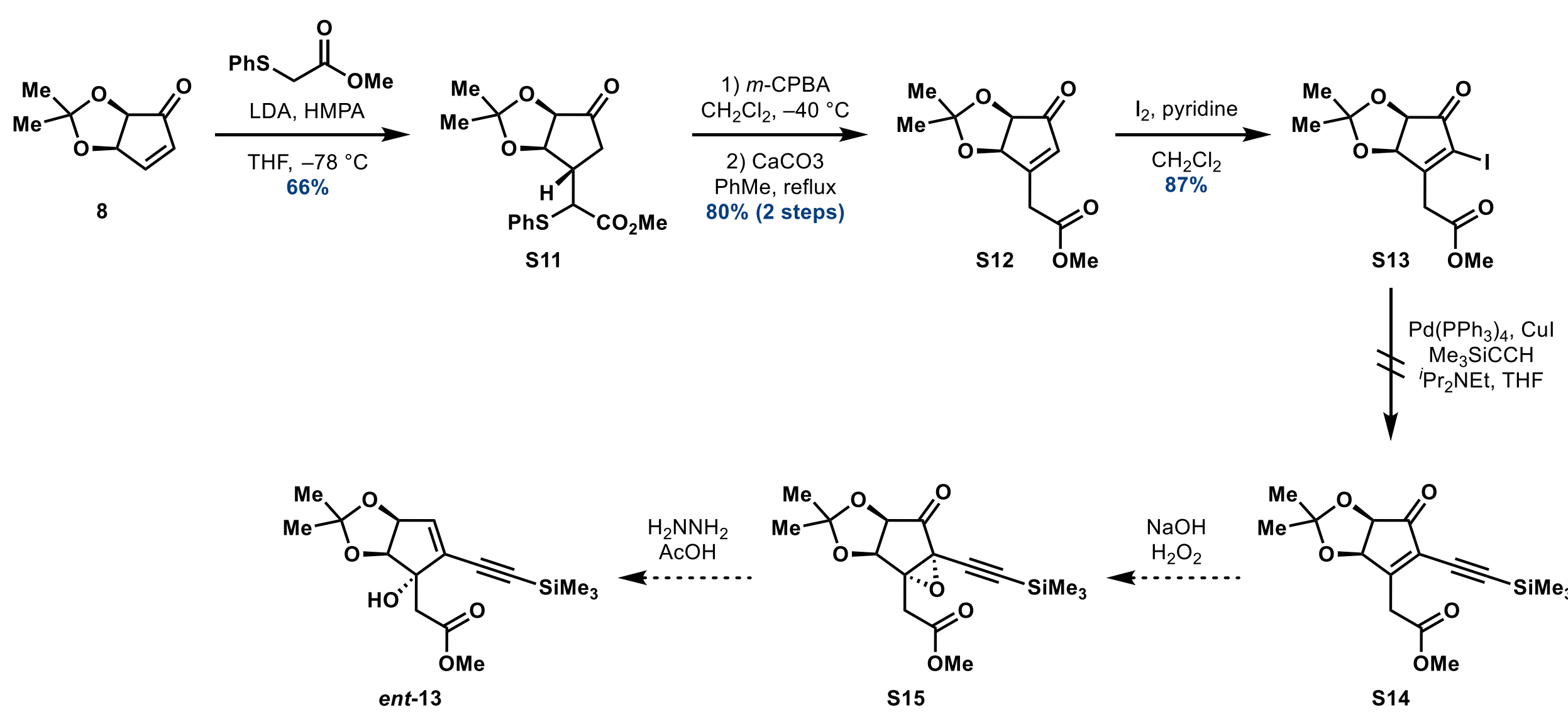

Scheme S4. Synthesis of **S13** from **8** via **S12**[1] in preparation for Weitz–Scheffer and Wharton reaction. Various attempts to synthesize **S14** via Sonogashira reaction failed due to decomposition of **S13**. LDA = lithium diisopropylamide; HMPA = hexamethylphosphoramide; *m*-CPBA = *meta*-chloroperoxybenzoic acid.

Synthesis | DOI 10.1055/a-2951-5568 | © The Author(s).

Optimization of 1,4-borylation/oxidation

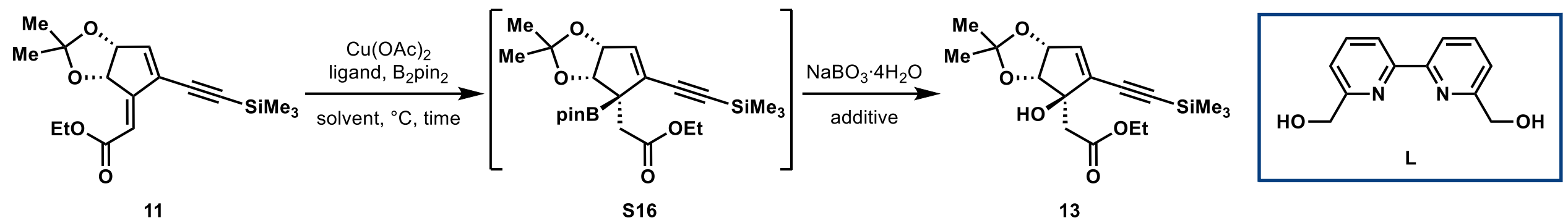

reaction optimizations[a]

Cu(OAc)$_2$	ligand	B$_2$pin$_2$	solvent	temperature	time	NaBO$_3$•4H$_2$O	additive	yield
5 mol%	N/A	1.2 equiv.	DMF/H_2O (2:1)	0 *to* 4 °C	18 h	8 equiv.	N/A	trace
10 mol%	N/A	1.6 equiv.	DMF/H_2O (1:1)	0 *to* 4 °C	18 h	8 equiv.	N/A	trace
10 mol%	**L** (12 mol%)	1.6 equiv.	THF (wet)	0 *to* 4 °C	18 h	8 equiv.	N/A	59%[b]
10 mol%	**L** (12 mol%)	1.6 equiv.	THF (wet)	0 *to* r.t.	18 h	8 equiv.	H_2O	43%[c]
15 mol%	**L** (18 mol%)	1.6 equiv.	THF (wet)	0 *to* r.t.	18 h	8 equiv.	THF/H_2O (3:2)[d]	30%
20 mol%	**L** (24 mol%)	1.6 equiv.	THF (wet)	0 *to* r.t.	18 h	3 equiv.	H_2O	53%
20 mol%	**L** (24 mol%)	1.6 equiv.	THF (wet)	r.t.	6 h	3 equiv.	H_2O	65%
20 mol%	**L** (24 mol%)	1.6 equiv.	THF (dry)	r.t.	18 h	3 equiv.	H_2O	n.r.
20 mol%	**L** (24 mol%)	1.6 equiv.	THF (wet)	r.t.	18 h	3 equiv.	H_2O	n.r.[e]

Table S3. Optimization details of 1,4-borylation/oxidation reaction from **11** to **13**. [a] All reactions were degassed with N_2 and under exclusion of light unless otherwise noticed. [b] 59% of **S16**, no detection of **13**. [c] 43% of alcohol with loss of SiMe$_3$ group. [d] Workup before oxidation. [e] Opened to air and not covered with aluminum foil. B$_2$pin$_2$ = Bis(pinacolato)diboron.

Model study of Sonogashira reaction with vinyl bromide 7

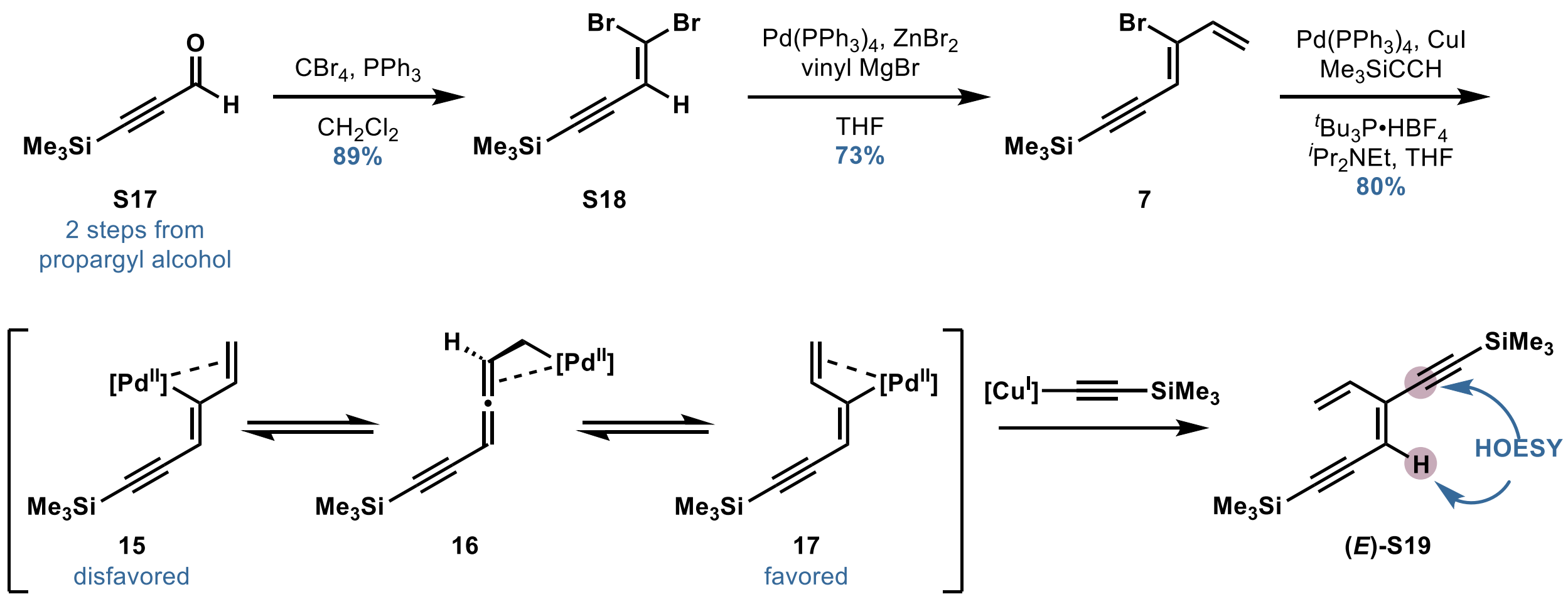

Scheme S5. Synthesis of **7** from **S17**[2] in preparation for the model Sonogashira reaction. Heteronuclear Overhauser Effect Spectroscopy (HOESY) was performed on **S19**. The alkene configuration was assigned to be (*E*) due to the correlation between the highlighted alkyne carbon and vinyl hydrogen.

Synthesis | DOI 10.1055/a-2951-5568 | © The Author(s).

Optimizations of Sonogashira fragment coupling

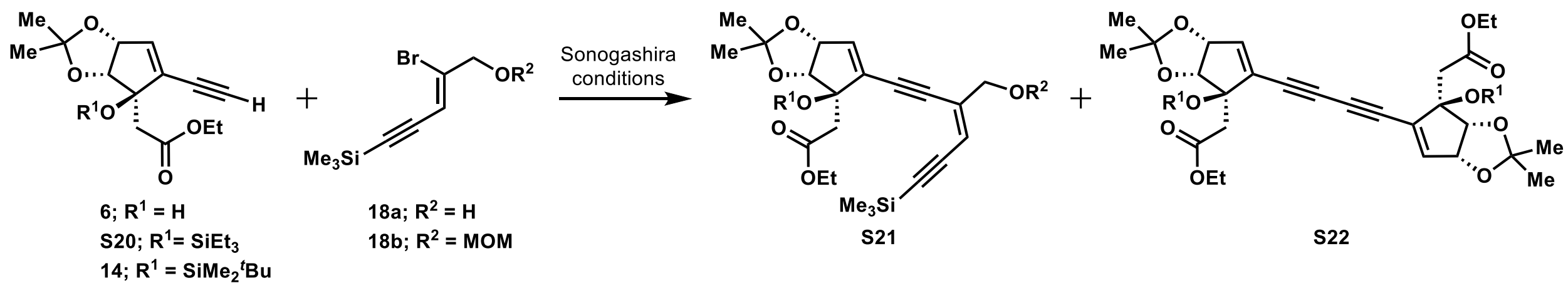

Sonogashira conditions

R groups	$Pd(PPh_3)_4$	CuI[a]	base	solvent	results
R^1 = H; R^2 = MOM	1 mol%	3 mol%	piperidine (4 equiv.)	PhMe (0.1 M)[b]	13% **S21**
R^1 = H; R^2 = MOM	5 mol%	30 mol%	$^i Pr_2NEt$ (5 equiv.)	THF (0.1 M)[b]	44% **S21**, 11% **S22**
R^1 = H; R^2 = MOM	5 mol%	6 mol%	$^i Pr_2NEt$ (5 equiv.)	THF (0.1 M)[b]	31% **S21**, 17% **S22**
R^1 = H; R^2 = H	5 mol%	30 mol%	$^i Pr_2NEt$ (5 equiv.)	THF (0.1 M)[b]	18% **S21**, 16% **S22**
R^1 = $SiEt_3$; R^2 = MOM	5 mol%	30 mol%	$^i Pr_2NEt$ (4 equiv.)<br>2,6-lutidine (2 equiv.)	DMF (0.1 M)[c,d]	51% **S21**, trace **S22**[e]
R^1 = $SiMe_2{}^tBu$; R^2 = MOM	5 mol%	30 mol%	$^i Pr_2NEt$ (4 equiv.)<br>2,6-lutidine (2 equiv.)	DMF (0.1 M)[b,d]	68% **S21**, trace **S22**
R^1 = $SiMe_2{}^tBu$; R^2 = MOM	5 mol%	30 mol%	$^i Pr_2NEt$ (4 equiv.)<br>2,6-lutidine (2 equiv.)	DMF (0.1 M)[c,d]	74% **S21**, trace **S22**

Table S4. Optimization details of Sonogashira fragment coupling. All reactions were performed under N_2 with exclusion of light. All results were reported with isolated yields unless otherwise noticed. [a] CuI was purified by washing with refluxing THF in Soxhlet extractor and stored under N_2. [b] Solvent was degassed by bubbling N_2. [c] Solvent was degassed by freeze-pump-thaw for three cycles. [d] 4Å MS (50 mg/mL) was added to the reaction. [e] Yields were estimated by ^1H NMR due to the decomposition of product upon workup and purification. MOM = methoxymethyl.

Synthesis | DOI 10.1055/a-2951-5568 | © The Author(s).

Attempts to obtain aldehyde 21a and 21b via oxidation

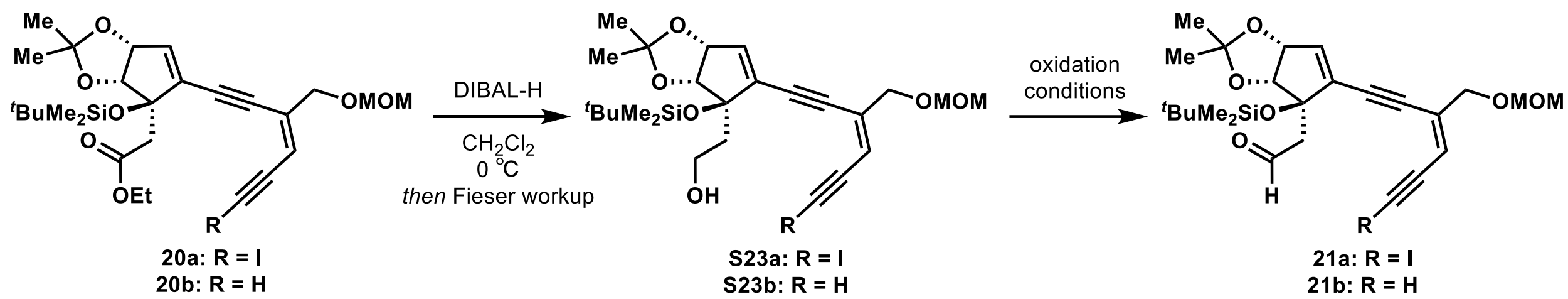

failed oxidation attempts

conditions	R = I	R = H
Dess–Martin periodinane $NaHCO_3$, CH_2Cl_2	94%[a]	91%[a]
TEMPO, PIDA, CH_2Cl_2	N/A	no product
[Cu(MeCN)$_4$]OTf, TEMPO bpy, NMI, MeCN, air	no reaction	not applicable
SO_3•pyridine, ^{i}Pr$_2$NEt DMSO, CH_2Cl_2, 0 °C	decomposition	decomposition
Ac_2O, DMSO, 30 °C	decomposition	decomposition
^{n}Pr$_4$NRuO$_4$, NMO, CH_2Cl_2	decomposition	decomposition
$(COCl)_2$, DMSO, NEt_3, CH_2Cl_2	decomposition	decomposition
Ag_2CO_3/Celite, PhH, reflux	decomposition	decomposition

Table S5. Failed oxidation attempts to obtain **21a** and **21b** without column chromatography. [a] Yield was determined via ^{1}H NMR integrations since product contains 2-iodobenzoic acid as impurity after multiple sat. aq. $NaHCO_3$ washes. DIBAL-H = diisobutylaluminum hydride; TEMPO = (2,2,6,6-tetramethylpiperidin-1-yl)oxyl; PIDA = phenyliodine(III) diacetate; bpy = 2,2'-bipyridine; NMI = *N*-methylimidazole; NMO = *N*-methylmorpholine *N*-oxide.

Synthesis | DOI 10.1055/a-2951-5568 | © The Author(s).

Condition screening for the ring-closing acetylide addition

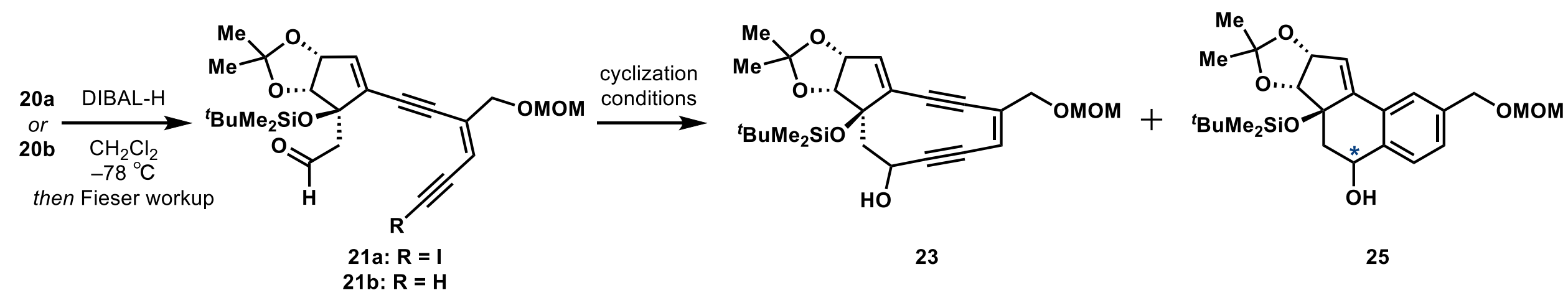

cyclization conditions

substrate	conditions	results
**21b**	KHMDS, THF	decomposition
**21b**	$Zn(OTf)_2$, NEt_3, 4Å MS<br>*N*-methylephedrine, PhMe	decomposition
**21b**	^{t}BuLi, THF, –78°C *to* r.t.	decomposition
**21a**	^{t}BuLi, THF, –78°C *to* r.t.	decomposition
**21a**	$NiCl_2$, $CrCl_2$, 4Å MS, THF	decomposition
**21a**	$NiCl_2$, $CrCl_2$, 4Å MS, THF[a]	50% of **25** over two steps[b]

Table S6. Condition screening for the ring-closing acetylide addition. [a] THF was freshly distilled from sodium/benzophenone. [b] No detection of **23** even if the reaction was quenched prematurely. DIBAL-H = diisobutylaluminum hydride; KHMDS = potassium bis(trimethylsilyl)amide. MS = molecular sieves.

Synthesis | DOI 10.1055/a-2951-5568 | © The Author(s).

References

1. Yang, M.; Schneller, S. W.; Korba, B. *J. Med. Chem.* **2005**, *48*, 5043.
2. Jung, W.-H.; Guyenne, S.; Riesco-Fagundo, C.; Mancuso, J.; Nakamura, S.; Curran, D. P. *Angew. Chem. Int. Ed.* **2008**, *47*, 1130.

Synthesis | DOI 10.1055/a-2951-5568 | © The Author(s).
